# Thonningianin A derived from *Penthorum chinense* Pursh alleviates cerebral ischemia/reperfusion-mediated apoptosis and pyroptosis through the activation of PINK1/Parkin-dependent mitophagy

**DOI:** 10.1186/s13020-025-01247-2

**Published:** 2026-01-16

**Authors:** Qianfang Yao, Guishan Hu, Can Yin, Anguo Wu, Guangqiang Hu, Dalian Qin, Xiaogang Zhou, Betty Yuen-Kwan Law, Xi Du, Li Chen, Jianqiao Li, Hong Lin, Xin Long, Jianming Wu, Lu Yu

**Affiliations:** 1 Sichuan Key Medical Laboratory of New Drug Discovery and Drugability Evaluation, School of Pharmacy, School of Basic Medical Sciences, Southwest Medical University, Luzhou, 646000 China; 2 Department of Anatomy, School of Basic Medical Sciences, Southwest Medical University, Luzhou, 646000 China; 3 State Key Laboratory of Quality Research in Chinese Medicine, Macau University of Science and Technology, Taipa, Macau China; 4 Department of Chemistry, School of Basic Medical Sciences, Southwest Medical University, Luzhou, 646000 China; 5 Department of Neurology, The Affiliated Traditional Chinese Medicine Hospital of Southwest Medical University, Luzhou, 646000 Sichuan China; 6 Clinical Medical College, Southwest Medical University, Luzhou, 646000 Sichuan China

**Keywords:** Cerebral ischemia/reperfusion injury, Apoptosis, Pyroptosis, Autophagy, Mitophagy, PINK1/Parkin signaling pathway, *Penthorum chinense* Pursh, Thonningianin A

## Abstract

**Background:**

Cerebral ischemia/reperfusion injury (CI/RI) remains a critical barrier to effective ischemic stroke (IS) treatment. While mitophagy activation has been shown to attenuate apoptosis and pyroptosis, thereby ameliorating CI/RI, the therapeutic potential of natural compounds targeting this pathway remains underexplored. *Penthorum chinense* Pursh (PCP), a traditional hepatoprotective herb, contains Thonningianin A (TA), a bioactive compound with reported autophagic properties. However, the role and mechanisms of TA in CI/RI mitigation remain unclear.

**Methods:**

In vivo, a middle cerebral artery occlusion/reperfusion (MCAO/R) rat model was established to evaluate TA’s neuroprotective effects via TTC staining, Longa neurological scoring, and immunofluorescence staining. In vitro, oxygen–glucose deprivation/reoxygenation (OGD/R)-treated HT22 and BV2 cells were used to assess TA’s impact on cell viability (MTT, Hoechst/PI staining), mitochondrial oxidative stress (DHE, TMRM, JC-1, Mito-Tracker staining and Western blot), apoptosis (flow cytometry, immunofluorescence staining, Hochest and PI staining and Western blot), and pyroptosis (EthD-2/YO-PRO-1 staining and Western blot). Autophagy and mitophagy modulation was investigated using rapamycin (Rap), 3-MA (autophagy inhibitor), CCCP (mitophagy inducer), and AC220 (mitophagy inhibitor) in EGFP-LC3-U87 and mCherry-GFP-FIS1-293T cells. Co-localization immunofluorescence and Western blotting were employed to validate PINK1/Parkin pathway involvement.

**Results:**

TA administration significantly improved neurological function, reduced cerebral infarct volume, and attenuated neuronal damage in MCAO/R rats. In vitro, TA suppressed OGD/R-induced mitochondrial oxidative stress and apoptosis in HT22 cells while mitigating pyroptosis in BV2 microglia. Mechanistically, TA activated PINK1/Parkin-dependent mitophagy, as evidenced by enhanced LC3-II/I ratio, and increased mitochondrial-autophagosome co-localization. Crucially, TA’s anti-apoptotic and anti-pyroptotic effects were abolished upon mitophagy inhibition. These findings were corroborated in the MCAO/R model, where TA upregulated PINK1/Parkin signaling and mitigated cell damage.

**Conclusion:**

This study identifies TA as a novel natural agent alleviating CI/RI by activating PINK1/Parkin-mediated mitophagy, thereby concurrently suppressing apoptosis and pyroptosis. These findings provide the first elucidating the molecular mechanis underlying TA's potential as a therapeutic candidate for IS.

**Graphical Abstract:**

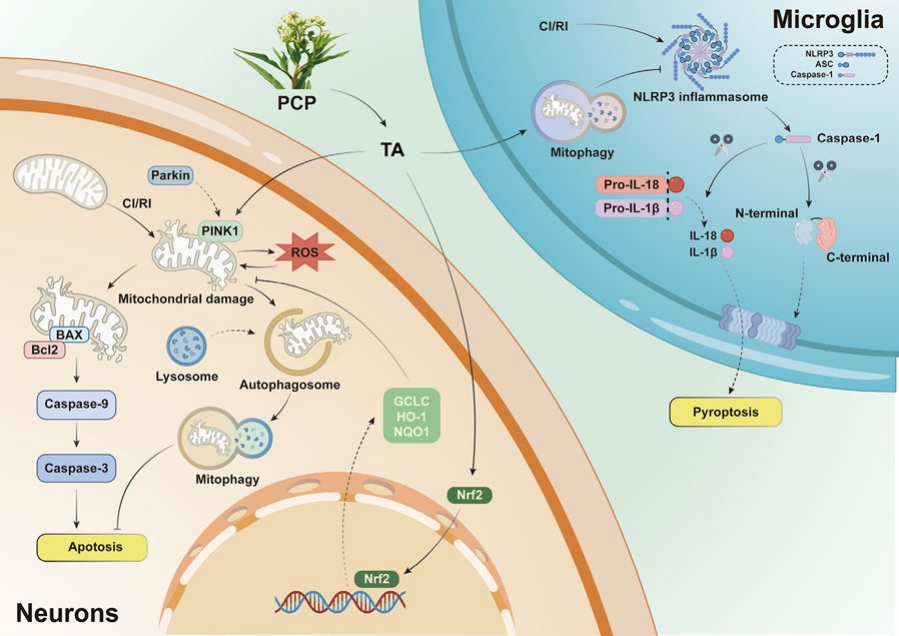

**Supplementary Information:**

The online version contains supplementary material available at 10.1186/s13020-025-01247-2.

## Introduction

Stroke is the second leading cause of death and the third leading cause of disability worldwide [1]. Ischemic stroke (IS) accounts for 67.3–80.5% of all stroke cases, imposing a significant economic burden on society [2]. The key treatment for IS is the timely restoration of blood flow, which is crucial for minimizing neuronal damage caused by ischemia. With the increasing adoption of intravenous thrombolysis and endovascular treatment techniques, the rates of vascular recanalization in acute IS have gradually improved [3]. However, patients face the risk of cerebral ischemia/reperfusion injury (CI/RI), posing a significant challenge to IS recovery.

Mitochondria play a central role in eukaryotic oxidative metabolism [4], and are critical mediators of neuronal cell death following ischemia [5]. Studies have demonstrated that focal brain ischemia and subsequent reperfusion trigger a cascade of events that activate mitophagy [6]. Following brain ischemia, blood flow dramatically decreases, resulting in hypoxia, hypoglycemia, abnormal mitochondrial structure, and disrupted oxidative phosphorylation [7]. During reperfusion, disrupted mitochondrial oxidative phosphorylation, increased reactive oxygen species (ROS), and impaired clearance capabilities create excessive oxidative stress, exacerbating mitochondrial damage and perpetuating the vicious cycle of CI/RI [8]. Therefore, the timely removal of damaged or dysfunctional mitochondria, while preserving functional ones, is crucial for protecting neuro from CI/RI stress. Mitophagy serves as an essential mechanism for maintaining mitochondrial quality and ROS balance through the degradation of damaged mitochondria [9]. Upon activation, mitophagy specifically identifies, targets, and degrades dysfunctional mitochondria. Additionally, ROS have been implicated in the activation of the NLRP3 inflammasome. During cerebral ischemia–reperfusion, mitophagy can inhibit cell apoptosis and pyroptosis by inhibiting the activation of the NLRP3 inflammasome, thereby exerting a neuroprotective function [10]. Consequently, the regulation of mitophagy presents a promising therapeutic strategy for the treatment of IS. PTEN-induced putative kinase 1 (PINK1) is a central mediator of mitophagy [11]. Mitochondria exhibiting poor structural integrity and low membrane potential activate PINK1, which then recruits Parkin, an E3 ubiquitin ligase, to the mitochondrial surface, ultimately triggering mitophagy. Accumulated evidence suggests that PINK1/Parkin-mediated autophagy is involved in the pathogenesis of various diseases, including stroke, neurodegenerative disorders, and multiple sclerosis [12, 13].

Significant number of active ingredients with anti-CI/RI properties have been isolated from herbal sources of Traditional Chinese medicines (TCMs). Notable examples encompass ginsenosides from Ginsenoside, angelica polysaccharides from Radix Angelica Sinensis, and astragaloside and polysaccharides from Astragalus [14], these compounds have demonstrated significant therapeutic effects on CI/RI in both preclinical and clinical studies. Recent research shows that Notoginsenoside R1 alleviates CI/RI through regulating the gastrointestinal axis [15]*,* Astragaloside IV mitigates CI/RI by inhibiting ferroptosis via activation of the P62/Keap1/Nrf2 pathway [16]. *Penthorum Chinense* pursh (PCP), a herbal plant from the PCP family, was first documented in the Ming Dynasty’s Materia Medica for Relief of Famines. It is well known for its antioxidant, anti-hepatitis B virus, and anti-inflammatory properties, as well as its potential to prevent and treat liver damage [17]. The liver and brain exhibit shared pathological mechanisms in response to injuries to the disease, particularly oxidative stress and inflammation [18, 19]. Tanshinone A (TA), a monomer derived from PCP, demonstrates potent antioxidant and anti-inflammatory effects, as evidenced by its therapeutic potential in neurodegenerative disorders such as Alzheimer’s disease (AD) and Parkinson’s disease (PD) [20–23]. In addition, it has proved that the active component EPC from PCP exerts protective effects on astrocytes subjected to oxygen–glucose deprivation and reperfusion (OGD/R) [47]. In our previous drug screening studies involving OGD/R, it was found that TA significantly restores the viability of HT22 cells post-OGD/R treatment, reduces ROS levels in HT22 cells, and diminishes NLRP3 protein levels in BV2 cells, all while exhibiting notable autophagic activity. Accordingly, current study aims to investigate whether TA activates mitophagy through the PINK1/Parkin signaling pathway, ultimately inhibiting cell apoptosis and pyroptosis, thereby improving the outcomes of CI/RI.

## Materials and methods

### Chemicals and reagents

PCP stems, flowers, and leaves were purchased from Zi Ning Zhong Yao Yin Pian Co., Ltd. (Chuan 20110407, Sichuan, China). TA (≥ 98% purity, HPLC) was isolated and identified from PCP in a previous study [20]. The positive control drug Nimodipine (NMDP) was purchased from Heowns Biochem LLC (66085-59-4, Tianjin, China). Rap (T1537), AC220 (T2066) and 3MA (T1879) were purchased from TargetMol (Shanghai, China). 2，3，5-Triphenyltetrazolium Chloride (TTC) (298-96-4) and Triton X-100 (9002-93-1) were purchased from Harvey Biotechnology (Beijing, China). Pentobarbital sodium (P11011), MTT (298-93-1) and Hoechst (23491-45-4) were purchased from Sigma (St. Louis, MO, USA). DMSO (67-68-5) was purchased from Aladdin (Shanghai, China), and Pancreatin (BL521A) was purchased from Biosharp (Chengdu, China). JC-1 (C2006-100), YO-PRO-1 (C2022) was purchased from Beyotime Biotechnology (Shanghai, China). Eth-D2 (E3599) was purchased from Thermo Fisher (MA, USA). AnnexinV-FITC (FXP145) was purchased from Beijing 4A Biotech Co., Ltd (Beijing, China). Tetramethylrhodamine methyl ester perchlorate (TMRM) (E2945-5) was purchased from Selleck (Shanghai, China), propidium iodide (PI) (FXP021-050) was purchased from 4A Biotech Co., Ltd (Shenzhen, China). Mito-tracker (C1035) was purchased from Beyotime (Shanghai, China). DHE (VB1-3028353-VC) was purchased from Invitrogen (California, USA). Lipomaster 3000 (7E732H3) transfection reagent was purchased from Invitrogen (Scotland, UK). Plasmids used in the study, including pmCherry-C1-ASC (PPL01752-2b), pGFP-N1-NLRP3 (PPL00151-2a), pEGFP-N1-caspase-1 (PPL00392-2e), pLVX-Puro-Tag RFP-GFP-fis1 (Mito-QC) (PPL01027-4a) were purchased from Public Protein/Plasmid Library (PPL, Nanjing, China). Antibodies including NLRP3 (15101S), IL-1β (12426S), IL-18 (57058), Nrf2 (12721S), BAX (14796S), Bcl2 (3498S), HO-1 (70081S) were purchased from Cell Signaling Technologies Inc (CST, Beverly, MA, USA). Antibodies against GSDMD (20770-1-Ap), Caspase-3 (25158-1-Ap), Caspase-9 (10380-1-Ap), NQO1 (11451-1-Ap), GCLC (12601-1-Ap), LC3 (14600-1-Ap), PINK1 (23274-1-Ap), Parkin (14060-1-Ap), Antibody Caspase-1 (22,915-1-ap), GAPDH (6004-1-Ig), β-actin (66009-1-Ig), CoraLite^®^ Plus 488 goat anti-rabbit IgG (RGAR002), CoraLite^®^ Plus 594-Goat Anti-Mouse IgG (RGAR002), were purchased from Proteintech Group, Inc (Wuhan, China). Antibodies ASC (SC-514414p) was purchased from Santa Cruz Biotechnology (CA, USA), Goat anti-Rabbit (09031120), Goat anti-Mouse (11020420) IgG Secondary Antibody, were purchased from Medical & Biological Laboratories Co., Ltd (MA, US).

### Animals

Adult male Sprague–Dawley rats (260 ± 20 g) were purchased from Chongqing Tengxin Biotechnology Company (Chongqing, China), and housed in constant room temperature (22 ± 2 °C) with 50 ± 10% humidity under a 12/12 h light/dark cycle with food and water ad libitum for at least 1 week before the experiments. All experiment procedures were conducted according to the Animal Care and Use Committee Guidelines of Southwest Medical University (No. 20240928-001). Every effort was made to avoid/alleviate animal pain and distress and minimize the number of animals used in the project.

### Construction of MCAO/R model in rats

Rats were randomly assigned to six groups, with eight rats per group: the sham operation group, the MCAO/R group, and the MCAO/R + TA treatment groups with different concentrations (0.25, 0.5, or 1 mg/kg) and positive control group with NMDP (10 mg/kg). The MCAO/R model was established using the thread embolism method. Prior to surgery, the rats were fasted and deprived of water for 12 h. Following established protocols from our previous research [24]. Anesthesia was administered via an intraperitoneal injection of Pentobarbital sodium (40 mg/kg), then a midline neck incision was made to isolate the right common carotid artery (CCA), internal carotid artery (ICA), and external carotid artery (ECA). The ECA was then ligated, and a prepared filament was inserted into the ECA stump near its bifurcation with the ICA. The filament was advanced 18–20 mm into the ICA until resistance was felt, inducing MCAO. Following 2 h of ischemia, rats were re-anesthetized, the filament was withdrawn to achieve reperfusion. Rats that died during surgery were excluded and replaced. Sham-operated rats underwent identical procedures excluding filament insertion.

### Neurobehavioral assessment

Neurological function was evaluated using the Longa scoring method at 24 h post-reperfusion: 0 points for asymptomatic; 1 point if the injured contralateral forelimb could not straighten when the tail was lifted; 2 points for rotation towards the injured side; 3 points for tilting towards the opposite side; and 4 points for inability to walk spontaneously or unconsciousness. Rats exhibiting abnormalities during the procedure were excluded.

### TTC staining

The rats were anesthetized and euthanized, and the brains were extracted and stored at −20 °C for 10 min. Coronal sections, each 2 mm thick, were cut between the frontal and occipital poles using a blade. The brain slices were then incubated in 0.5% TTC solution at 37 °C in the dark for 20 min. Viable brain regions appeared red, while infarcted areas were white. The infarct area and total area for each slice were measured using ImageJ software, the brain infarct rate is equal to infarct volume divided by total volume.

### Cell culture and establishment of OGD/R cell model

HT22 and BV2 cells were cultured in DMEM (Gibco, United States) with 10% FBS (Procell). U87 cells were cultured in MEM (Gibco, United States) with 10% FBS. Cells were maintained at 37 °C in an incubator containing 5% CO_2_.

To establish the oxygen–glucose deprivation/reoxygenation (OGD/R) cell model, the complete medium was removed from each group of cells and replaced with glucose-free medium to simulate ischemic conditions, the cells were then placed in a hypoxic chamber filled with 95% N_2_ and incubated for 10 h (HT22) or 8 h (BV2) to induce hypoxia. Following this period, the cells were removed from the hypoxic environment, the glucose-free medium was replaced with a complete medium, and the cells were incubated for an additional 24 h to allow for reoxygenation and glucose recovery. During the modeling process, different concentrations of TA (1.25 μM, 2.5 μM and 5 μM) were administered.

Furthermore, HT22 cells were cultured with BV2 supernatant prepared as follows: BV2 cells were seeded in 96-well plates at a density of 4 × 10^3^ cells per well in 100 μL medium and incubated at 37 °C for 24 h. Subsequently, TA was administered, and the BV2 cells were subjected to OGD for 8 h to establish the model. Following OGD, the cells were restored to normoxic conditions with complete medium for 24 h. The supernatant was then collected, centrifuged at 1000 × g for 10 min to remove cell debris, and applied to HT22 cells for 24 h of culture. As a control, HT22 cells were treated with supernatant collected from BV2 cells maintained under normoxic conditions throughout the experiment.

### MTT detection

HT22 and BV2 cells in the logarithmic growth phase were trypsinized, centrifuged, and gently resuspended. The cells were then seeded into a 96-well plate at a density of 4 × 10^3^ cells per 100 μL. The cells were incubated in an incubator at 37 °C for 24 h. Following OGD/R and TA treatment, a mixture of 5 mg/mL MTT solution and complete culture medium was prepared, and 100 μL of this mixture was added to each well of the 96-well plate. The plates were incubated for an additional 2 h, the medium containing MTT was aspirated, and 100 μL of DMSO was added to each well. The plates were shaken gently for 10 min to ensure complete solubilization of the formazan crystals. Finally, the absorbance of each well was measured at a detection wavelength of 570 nm, and cell viability was calculated using the appropriate formula.

### Hoechst/PI staining

HT22 cells were subjected to OGD and treated with various concentrations of TA for 10 h, the cells were reoxygenated for 24 h and subsequently treated with different concentrations of TA for an additional 24 h. Afterward, the medium was removed, and the cells were fixed with 4% paraformaldehyde. A complete medium containing Hoechst and PI dyes was then added, and the cells were incubated in the dark for 15 min. Finally, images were captured and observed using an inverted fluorescence microscope, and quantitative analysis was performed using ImageJ software.

### Mitochondrial membrane potential staining

Mitochondrial membrane potential (MMP) was assessed using a JC-1 kit or Tetramethylrhodamine methyl ester perchlorate (TMRM) fluorescent dye. HT22 cells were subjected to OGD/R and TA treatment. Subsequently, the cells were incubated with JC-1 at a concentration of 2 μg/mL or TMRM at 50 nM at 37 °C in the dark for 20 ~ 30 min. For the JC-1 dye, red fluorescence in the mitochondrial region indicates the aggregation of concentrated JC-1, whereas green fluorescence signifies mitochondrial membrane depolarization, reflecting the presence of JC-1 in its monomeric form. For TMRM, it typically enters the mitochondrial matrix, displaying strong red or orange fluorescence. When the integrity of the mitochondrial membrane is compromised, TMRM is released into the cytoplasm, leading to reduced fluorescence, which indicates a decrease in mitochondrial membrane potential. The images were subsequently captured and analyzed using a High-Intensity Imaging System microscope. The fluorescence intensities were quantified using ImageJ software.

### Mito-tracker staining

To evaluate mitochondrial morphology and network integrity, HT-22 cells were stained with Mito-Tracker Red and observed by confocal microscopy. Briefly, HT22 cells were subjected to OGD/R and TA treatment at a density of 1 × 10^5^ cells per dish, the cells were incubated with Hoechst reagent at 37 °C for 15 min, then were washed twice with PBS and incubated with 200 nM Mito-Tracker ™ Red CMXRos in the dark at 37 °C for 30 min and were fixed with 4% paraformaldehyde. Obtain images by using a confocal laser scanning microscope. For quantitative analysis, the mitochondrial skeleton was extracted using ImageJ software with the mitochondrial network analysis plugin.

### Flow cytometry

#### Detection of intracellular ROS content

After OGD/R treatment with or without TA intervention, HT22 cells were collected and centrifuged 12,000 rpm for 5 min the supernatant was then removed, 500 µL of 5 µM Dihydroethidium (DHE) solution was added to cells and incubated in a 37 °C incubator for 20 min, recentrifuged and mixed with PBS gently, then the cells were analyzed by a FluorSave™ mounting media (Calbiochem, San Diego, CA, USA). Data acquisition and analysis were performed by FlowJo 7.6.1 software (TreeStar, San Carlos, CA, USA).

#### Detection of cell apoptosis

Cell apoptosis of HT22 cells was measured by flow cytometry using the Annexin V-FITC/PI staining kit (BD, Biosciences, San Jose, CA, USA). In brief, cells were collected and centrifuged, and then a mixture solution containing 1 μL of AnnexinV-FITC and 2 μL of PI was added to the Annexin V solution and incubated for 20 min in the dark. Next, 100 μL of cell suspension was put into the flow tube and mixed gently, and then the cells were analyzed by a FluorSave^™^ mounting media (Calbiochem, San Diego, CA, USA). Data acquisition and analysis were performed by FlowJo 7.6.1 software.

### YO-PRO-1 and Eth-D2 staining

The pyroptosis of BV2 induced by OGD/R were detected by Eth-D2 and YO-PRO-1 staining. BV2 cells were treated with OGD/R and TA. Subsequently, the cells were incubated with 1 μM YO-PRO-1 and 1 μM Eth-D2 at 37 °C in the dark for 20 to 30 min. Subsequently, images were collected and analyzed using a high-intensity imaging system microscope. Fluorescence intensity was quantified using ImageJ software.

### Plasmid transfection

BV2 cells were planted into 6-well plates or 12-well plates with slides for 24 h, EGFP-N1-NLRP3, mCherry-C1-ASC, EGFP-N1-caspase-1, EGFP-N1-GSDMD or Mito-QC plasmids were transiently transfected into BV2 or HT22 cells with Lipomaster 3000 transfection reagent. After 24 h, OGD/R was performed and TA was added. Fluorescence intensity was observed and images were captured with an inverted fluorescence microscope.

### Western blot

Total or nuclear proteins extracted from cells or rat brain tissue were collected and protein concentration was measured. The protein solutions were subsequently loaded into the wells of an SDS-PAGE gel for electrophoresis and separation. After separation, the proteins were transferred to pre-activated PVDF membranes. The membranes were blocked with 10% nonfat milk in PBST and then incubated with the primary antibody overnight at 4 °C. Following this, the membranes were incubated with the corresponding secondary antibody at room temperature for 1 h. Protein bands were detected using ECL reagents and visualized with gel imaging equipment. Band intensity was quantified using ImageJ 6.0 software (National Institutes of Health, Bethesda, MD, United States).

### Immunofluorescence staining

The treated cells on the slides were fixed with 4% paraformaldehyde for 15 min. Paraffin sections of the brain tissue were dewaxed. Then the cells or brain sections were incubated with 0.5% Triton X-100 solution prepared in PBS at room temperature for 20 min to permeabilize the membranes. Subsequently, a blocking solution containing 5% BSA was added to the slides incubating for 30 min at room temperature. The primary antibody (NLRP3 and LC3, 1:1000 in cells, 1:200 in brain tissue), diluted in 5% BSA, was added and incubated overnight at 4 °C. The following day, the corresponding fluorescent secondary antibody was applied and incubated in the dark at room temperature for 1.5 h. Afterward, an appropriate volume of DAPI was added for nuclear staining in the dark for 5 min. The slides were then fixed with a FluorSavem Reagent. Fluorescence immunoimages were collected using the confocal laser scanning microscope (LSM 880; Zeiss, Oberkochen, Germany), and quantitatively analyzed using ImageJ 6.0 software.

### Statistical analysis

The data are presented as the means ± standard deviation (SD) and were analyzed using GraphPad Prism 8.0 software (San Diego, CA, USA). Differences between groups were compared using one-way ANOVA analysis followed by Tukey’s post hoc test. *p* < 0.05 was considered statistically significant. All experiments were independently repeated three or more times.

## Result

### TA improves neurological function damaging in MCAO/R rats

**Fig. 1:**
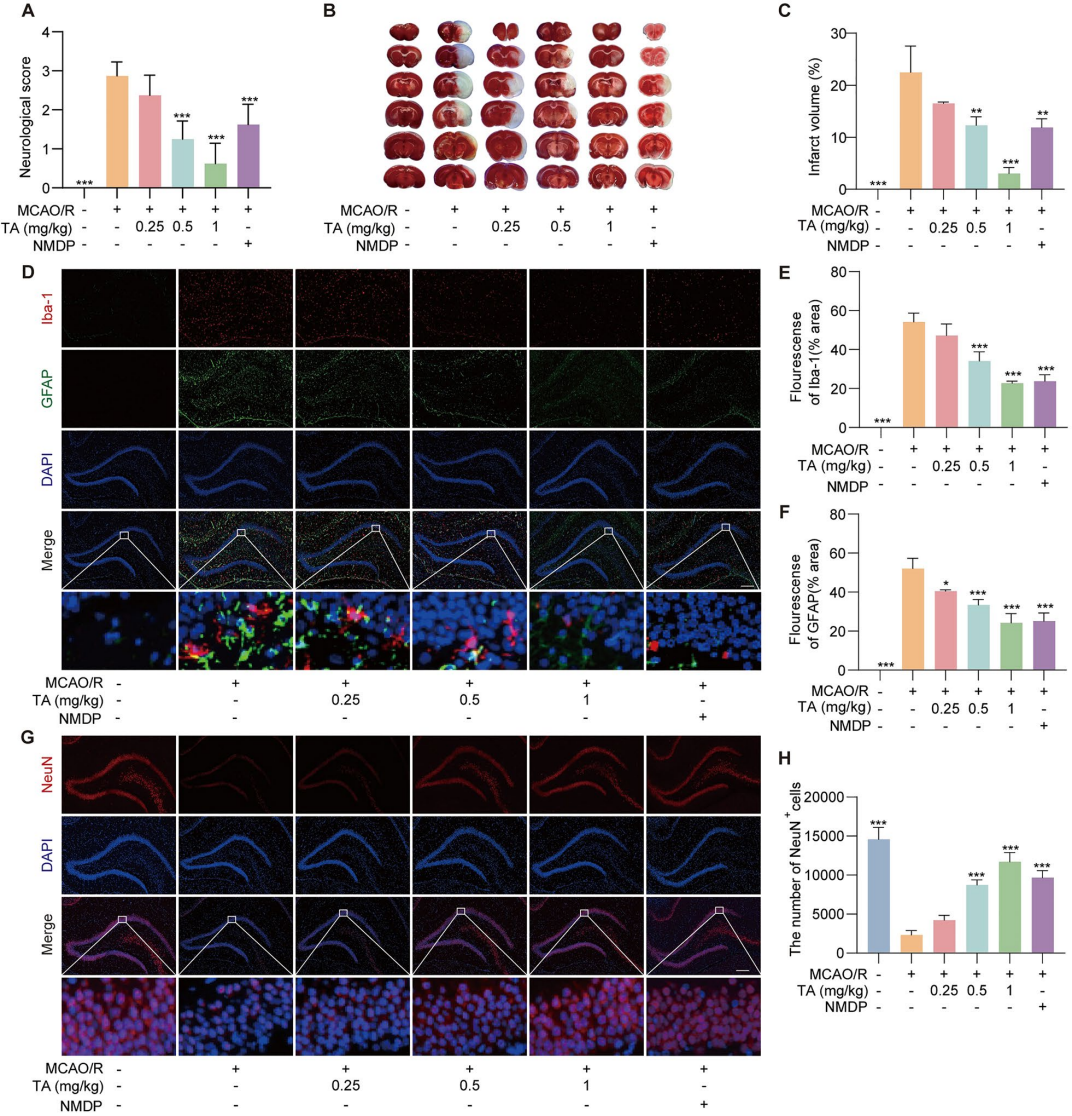
TA improved neurological function damaging in MCAO/R rats. **A** Neurological deficits were evaluated by using Zea-Longa Score at 24 h post-MCAO/R. The bar chart indicates the Neurological score of rats in different group. ***p < 0.001 versus MCAO/R alone group, n = 8. **B** Representative TTC-stained images showing infarct volume of rats at 24 h post-MCAO/R. **C** The bar chart indicates the cerebral infarct volume of rats in different group, **p < 0.01 and ***p < 0.001 versus the MCAO/R alone group, n = 5. **D** Representative immunofluorescence images of Iba-1^+^ microglia and GFAP^+^ astrocytes in the hippocampus region of rat brain. **E**, **F** The bar charts indicate quantified Iba-1 and GFAP immunofluorescence staining. *p < 0.05 and ***p < 0.001 versus MCAO/R alone group, n = 3. **G** Representative immunofluorescence images of NeuN in rat brain. **H** The bar chat indicates the number of NeuN's positive cells. ***p < 0.001 versus MCAO/R alone group, n = 3. Magnification: × 10; scale bar: 100 μm

To validate confirm the neuroprotective effects of TA on CI/RI, we employed the Longa scoring method to evaluate the neurological function and applied TTC staining to determine cerebral infarct volume change in MCAO/R rats with or without TA treatment. As shown in Fig. 1A, compared with the sham-operated group, the neurobehavioral scores increased in the rats of the MCAO/R group, while the indicated concentrations (0.25, 0.5 and 1 mg/kg) of TA and NMDP (10 mg/kg) were shown to reduce the neurobehavioral scores of rat. Subsequently, as shown in Fig. 1B, C, the TTC staining result showed that TA and NMDP decreased the cerebral infarct volume in MCAO/R rats. And, the effects of TA and NMDP on the proliferation and swelling of microglia and astrocytes and the recovery of neurons in MCAO/R rats were observed by immunofluorescence staining. The result indicates that there was obvious abnormal proliferation and swelling in astrocytes and microglia of rat brains in the MCAO/R alone group. However, TA and NMDP intervention significantly reduced the number and improved hypertrophy of astrocytes and microglia (Fig. 1D–F), and significantly increased the number of NeuN in the hippocampus region of the brain (Fig. 1G, H). All these results indicate that TA improves neurological function damage in MCAO/R rats.

### TA improves mitochondrial oxidative damage induced by OGD/R in HT22 cells

The cytotoxic concentration of TA in HT22 cells was assessed by using an MTT assay. The results are shown in Fig. S1, no significant toxicity was observed in HT22 cells at TA concentrations below 31.3 μΜ. The effect of varying durations of OGD, from 2 to 12 h, followed by 24 h of recovery with oxygen and glucose, cell viability was assessed using the MTT assay. The results are presented that the HT22 cell's survival rate was approximately 50% at 10 h of OGD followed by 24 h of recovery (Fig. S2). Therefore, this condition was chosen as the standard for the OGD/R model for subsequent experiments and recovery of HT22 cell viability was observed when TA was given under this condition (Fig. S3).

**Fig. 2:**
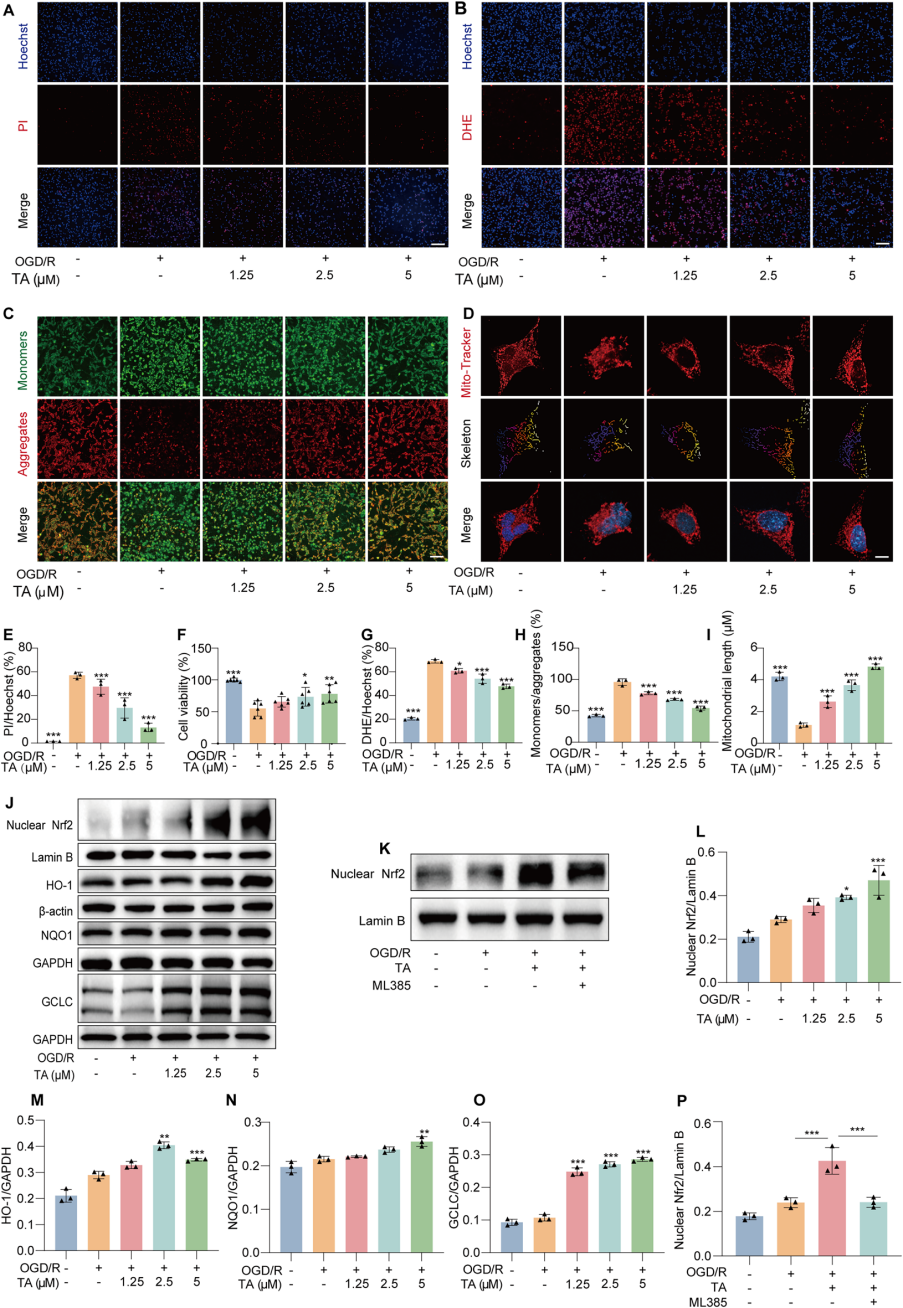
TA improved mitochondrial oxidative damage.** A** The death rate of HT22 cells was measured by Hoechst/PI staining in HT22 cells after 24 h of OGD/R induction and TA treatment. Magnification: 10 × , scale bar: 200 μm. **B** Representative images showing ROS release (DHE staining) and viable cells (Hoechst staining) in HT22 cells following OGD/R with or without TA treatment, × 10, scale bar: 200 μm. Bar chart indicates the rate of PI/Hoechst. *** p < 0.001 versus OGD/R alone group, n = 3. **C** Representative JC-1 staining images depict MMP in HT22 cells following OGD/R with or without TA treatment. Red fluorescence indicates JC-1 aggregates, while green fluorescence indicates the monomeric form. Magnification: 10 × , scale bar: 200 μm. **D** HT22 cells underwent OGD/R and TA treatment, followed by incubation with Hoechst reagent and Mito-Tracker ™ Red CMXRos to stain nuclei and mitochondria. Images were acquired using a confocal laser scanning microscope. Magnification: 64 ×, scale bar: 5 μm. **E** The bar chart indicates the DHE/Hoechst rate of HT22 cells. *p<0.05 and ***p < 0.001 versus OGD/R alone group, n = 3. **F** After 24 h of OGD/R induction and TA treatment, HT22 cell viability was measured by MTT assay, the result is as shown in the bar chart. *p < 0.05, **p < 0.01 and ***p < 0.001 , and versus OGD/R group, n = 6. **G** The bar chart indicates the DHE/Hoechst rate of HT22 cells. ***p < 0.001 versus OGD/R alone group, n = 3.** H** The bar chart illustrates the ratio of JC-1 aggregates to monomers in HT22 cells. ***p < 0.001 versus OGD/R alone group, n = 3. **I** The bar chart indicates mitochondrial length of HT22 cells in different group. ***p < 0.001 versus OGD/R alone group, n = 3. **J** Detection of nuclear Nrf2, HO-1, NQO1, and GCLC antioxidant protein expression by Western blotting. **K** HT22 cells subjected to OGD/R with or without TA, or TA + ML385 (an Nrf2 inhibitor), Western blotting was used to detect Nrf2 protein expression levels. **L**–**O** The bar chart indicates the ratios of nuclear Nrf2/LaminB, HO-1/β-actin, NQO1/GAPDH, GCLC/GAPDH. *p < 0.05, ** p < 0.01 and ***p < 0.001 versus OGD/R alone group. **P** The bar chart demonstrates that TA treatment enhanced OGD/R-induced nuclear Nrf2 expression, an effect reversed by the Nrf2 inhibitor ML385. ***p < 0.001 versus OGD/R group or OGD/R + TA group

Hoechst/PI staining results showed that TA significantly reduced the death rate of HT22 cells caused by OGD/R (Fig. 2A, E), and this conclusion was verified by the MTT experiment (Fig. 2F). Mitochondria serve as the primary sites for aerobic respiration in cells, and the excessive release of ROS from mitochondria is a critical factor contributing to CI/RI [25]. DHE, a premier fluorescent sentinel for superoxide anions, provides a sensitive and reliable means to monitor ROS dynamics within cells. The intensified red fluorescence observed in the OGD/R alone group illustrates a robust elevation of ROS levels. Conversely, the gradual attenuation of this red fluorescence signature upon TA treatment underscores a dose-dependent inhibitory effect of TA on OGD/R-induced ROS generation (Fig. 2B, G). As well as further corroborated by flow cytometry data (Fig. S7). To investigate the effect of TA on mitochondrial oxidative damage, we initially evaluated the MMP by employing the JC-1 staining technique. In the presence of high MMP, JC-1 accumulates within the mitochondrial matrix, forming polymers that subsequently emit a distinctive red fluorescence. Conversely, when the MMP is low, JC-1 exists in a monomeric state, which manifests as a green fluorescence. As shown in Fig. 2C, H, compared to the control group, the OGD/R alone group displayed a decrease in red fluorescence and an increase in green fluorescence, indicating a reduction in MMP and depolarization of the cells. In contrast, following TA intervention, a decline appeared in the green/red fluorescence ratio. This suggests that TA enhanced the MMP that is diminished by OGD/R. Furthermore, we utilized MitoTracker Red CMXRos (Fig. 2D, I), a red fluorescent probe that selectively binds to the active mitochondrial membrane, emitting red fluorescence signals that reflect the quantity, morphology, distribution, and movement of mitochondria, as well as their functional state and metabolic activity. The results demonstrated that TA improved the recovery of mitochondrial skeleton chain integrity disrupted by OGD/R in a dose-dependent manner. Nrf2, a master regulator of antioxidant defenses, orchestrates a complex network of genes to maintain cellular redox balance and shield against oxidative insults. Western blot analysis illuminated a significant upregulation of Nrf2 and its downstream effectors, heme oxygenase-1 (HO-1), NADPH quinone oxidoreductase 1 (NQO1), and glutamate-cysteine ligase catalytic subunit (GCLC), in response to OGD/R stress, and TA decreased the expression of all these protein (Fig. 2J, L–O). To unveil the precise mechanism underpinning TA's antioxidative prowess, the nuclear translocation of Nrf2, a pivotal event in its activation was detcected by Western blot analysis in HT22 cells, as presented in Fig. 2K, P, incontrovertibly demonstrated that TA fosters Nrf2's nuclear translocation, and the effect can be reversed by the Nrf2 inhibitor ML385. This finding underscored the critical role of Nrf2 nuclear translocation in mediating TA's ability to quell OGD/R-induced oxidative stress damage.

### TA significantly inhibited OGD/R-induced HT22 cell apoptosis

**Fig. 3:**
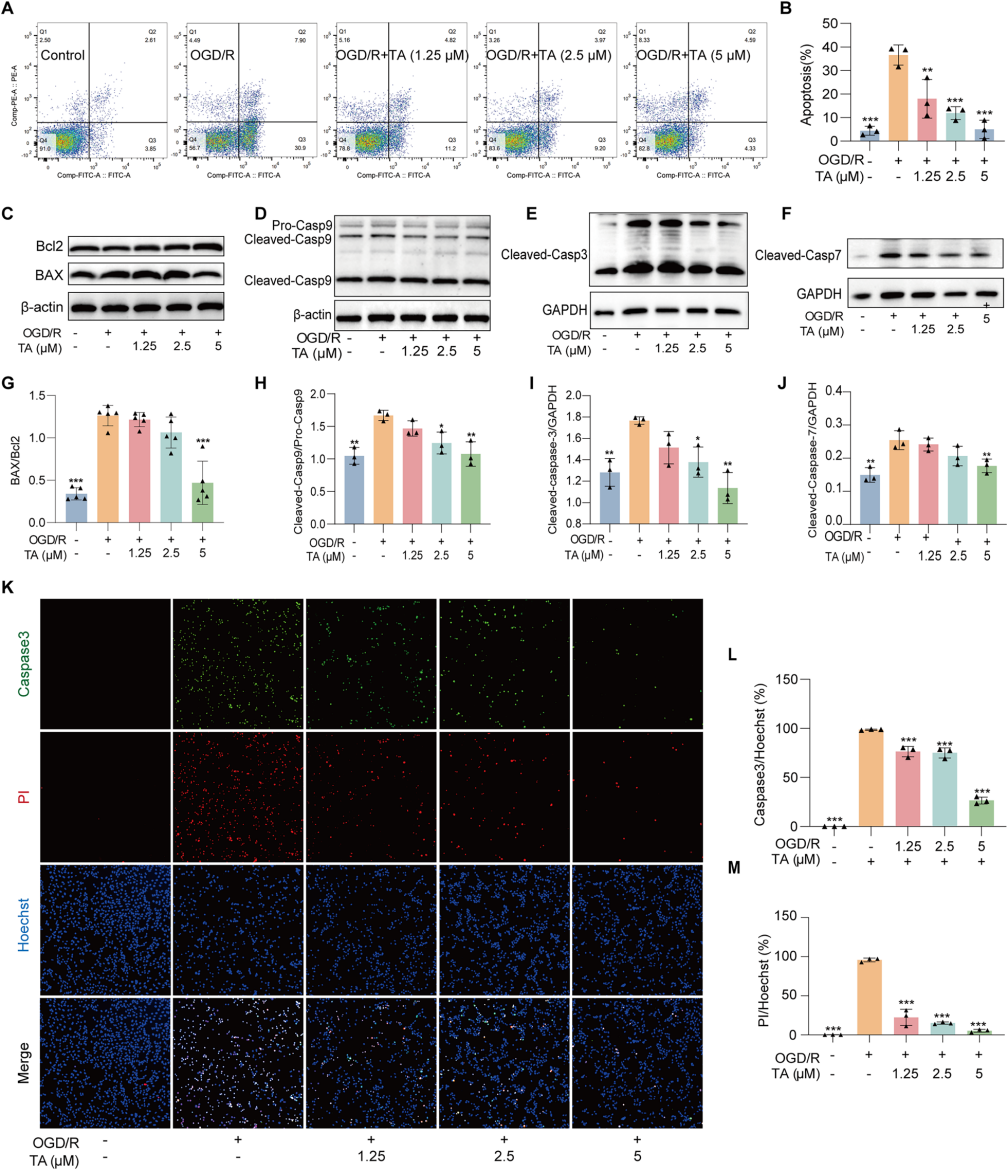
TA significantly inhibited OGD/R-induced HT22 cell apoptosis. **A** The cell apoptosis rate of HT22 cells was detected by flow cytometry methods. **B** Bar chart indicated that TA significantly reduced the apoptosis rate of HT22 cells induced by OGD/R. Compared to the alone OGD/R group, **p < 0.01 and ***p < 0.001. **C**–**F** The Western blotting detection of the protein expressions of Bax, Bcl2, Caspase-9, Caspase-3 and Caspase-7 in HT22 cells. **G**–**J** The bar chart demonstrated that TA significantly reduced the ratios of Bax/Bcl-2 (n = 5), Cleaved-Caspase-9/Pro-Caspase-9 (n = 3), Cleaved-Caspase-3/GAPDH (n = 3), and Cleaved-Caspase-7/GAPDH (n = 3). Compared to the alone OGD/R group, *p < 0.05, **p < 0.01, and ***p < 0.001. **K** BV2 cells were subjected to OGD/R and treatment with or without TA, the supernatants from these cells were then collected and used to treat HT22 cells. Subsequently, immunofluorescence staining was performed using Caspase-3, Hochest and PI staining, representative images were captured. Magnification, × 10; scale bar: 200 μm. **L**,**M** The bar chart indicates the rate of Caspase-3/Hochest, PI/Hochst. Compared with the alone OGD/R group, ***p < 0.001

Mitochondrial oxidative stress plays a pivotal role in regulating cell survival and apoptosis. Initially, the apoptosis of HT22 cells was evaluated by Annexin V-FITC flow cytometry analysis. Annexin V-FITC is a fluorescently labeled protein that specifically binds to membrane phosphatidylserine within the lipid bilayer, emitting green fluorescence. In the late stages of apoptosis, PI penetrates the nucleus, emitting red fluorescence. As illustrated in Fig. 3A, B, the apoptosis rate in the OGD/R group was significantly elevated compared to the control group, in contrast, treatment with TA markedly reduced the apoptosis rate induced by OGD/R. Subsequently, Western blot analysis revealed that the BAX/Bcl-2 ratio was significantly increased, and the cleaved forms of Caspase-9, Caspase-3 and Caspase-7 were elevated in the OGD/R group (Fig. 3C–J), indicating the occurrence of apoptosis. Importantly, treatment with TA significantly reversed the result, suggesting that TA effectively inhibits apoptosis induced by OGD/R. To further clarify the relationship between inflammation and apoptosis, HT22 cells were cultured with a supernatant of OGD/R-induced BV2 cells, Caspase3 staining showed that TA inhibited HT22 cell apoptosis mediated by inflammatory factors secreted by BV2 cells (Fig. 3K, L). Collectively, these findings provide compelling evidence that TA significantly inhibited OGD/R-induced apoptosis in HT22 cells.

### TA significantly inhibited OGD/R-induced pyroptosis in BV2 cells

Inflammation-related cell death has received considerable attention, Microglial cells are immune cells of the central nervous system. Firstly, the cytotoxicity of TA in BV2 cells was evaluated (Fig. S4) and the treatment conditions of the OGD/R model were evaluated in Fig. S5), the same as HT22 cells, 8 h of OGD followed by 24 h of recovery was chosen as the standard for the OGD/R model for subsequent experiments. It was observed that TA can significantly inhibit OGD/R-induced BV2 cell death by microscope (Fig. S6).

**Fig. 4:**
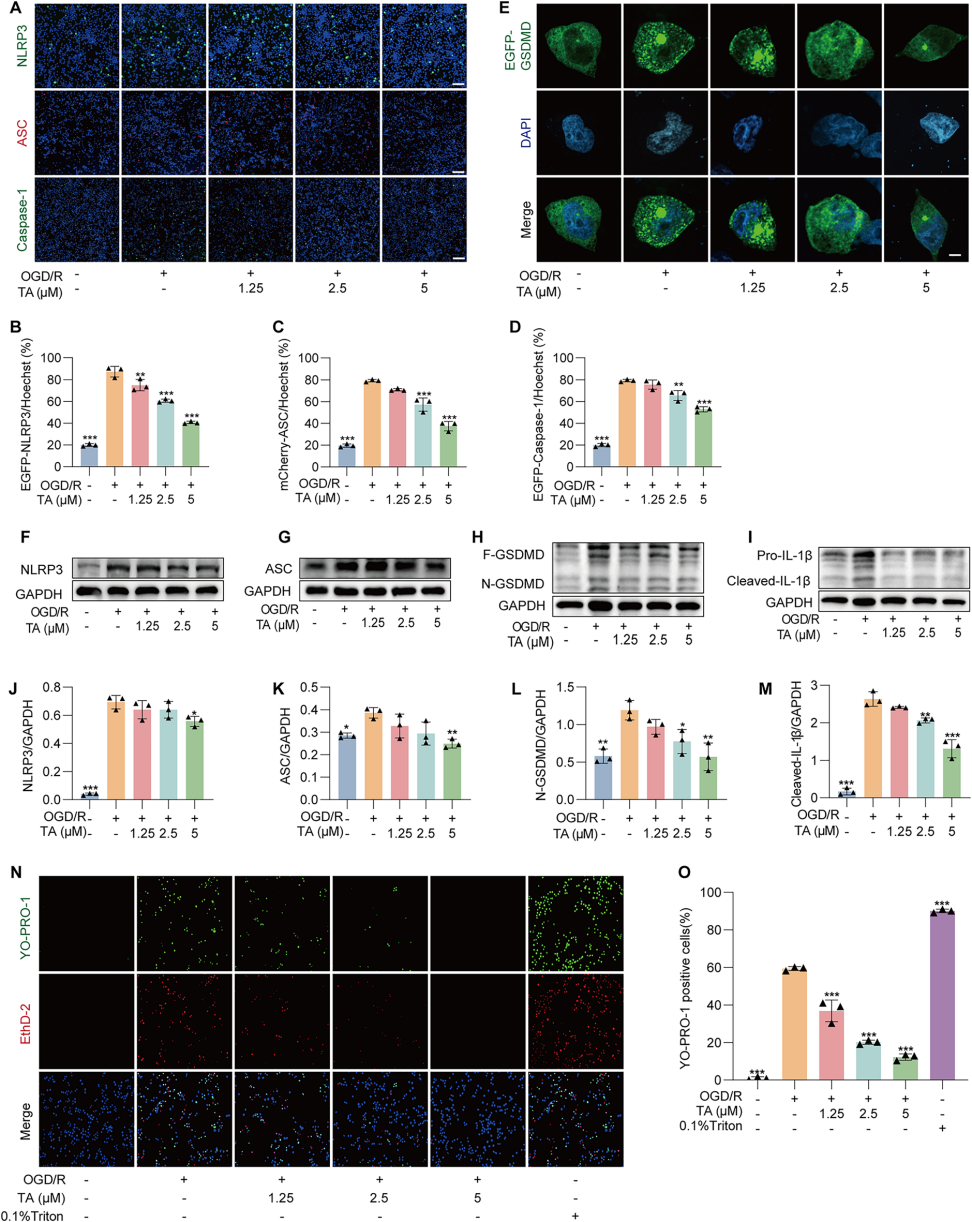
TA significantly inhibited OGD/R-induced pyroptosis in BV2 cells. **A** After transiently transfecting plasmids EGFP-N1-NLRP3, mCherry-C1-ASC, EGFP-N1-caspase-1, the cells underwent OGD/R with or without TA treatment, followed by Hoechst staining and imaging. Magnification: × 10; scale bar: 200 μm. **B**–**D** The bar chart presents the ratios of EGFP-NLRP3/Hoechst, mCherry-ASC/Hoechst, and EGFP-Caspase-1/Hoechst in different cell groups. **p < 0.01 and ***p < 0.001 versus OGD/R alone group, n = 3. **E** Representative images of fluorescence expression following transiently transfecting with EGFP-GSDMD plasmid and counterstaining with DAPI. Magnification: 64 × , scale bar: 5 μm. **F**–**I** The Western blotting detection of the protein expressions of NLRP3, ASC, GSDMD, and IL-1β in BV2 cells subjected to OGD/R, with or without TA treatment. **J**–**M** The bar chart presents the ratios of NLRP3/GAPDH, ASC/GAPDH, N-GSDMD/GAPDH and Cleaved-IL-1β/GAPDH. * *p* < 0.05, **p < 0.01 and ***p < 0.001versus OGD/R group, n = 3. **N** Representative images of Eth-D2/YO-PRO-1 staining in OGD/R-induced and TA or Triton-treated BV2 cells. Magnification: 10 × , scale bar: 200 μm. **O** The bar chart indicates the rate of YO-PRO-1 positivity cells. ***p < 0.001 versus OGD/R alone group

Excessive ROS activate the NLRP3 inflammasome, leading to the cleavage of gasdermin D (GSDMD) and the onset of pyroptosis. Plasmid EGFP-N1-NLRP3, mCherry-C1-ASC, EGFP-N1-caspase-1, and EGFP-N1-GSDMD were transiently to BV2 cells, and their expression was observed and captured by laser confocal microscope. Figure 4A–D demonstrated that, compared to the control group, the expression levels of ASC, NLRP3, Caspase-1, and GSDMD were significantly elevated following OGD/R treatment. However, following TA intervention, fluorescence intensity was notably decrease. Subsequently, OGD/R-induced BV2 cells were stained with GSDMD antibody after TA treatment. As shown in Fig. 4E, green fluorescent spots appeared in the vicinity of the BV2 cell membrane, indicating that GSDMD was activated and began to aggregate to the cell membrane, whereas the decrease in the champion spots near the cell membrane after TA treatment, indicating that TA could inhibit GSDMD activation and aggregation. The expression levels of pyroptosis-related proteins were assessed by Western blotting, TA significantly reduced the expression of NLRP3, ASC, N-GSDMD, and Pro-IL-1β (Fig. 4F–M). In conclusion, these findings suggest that TA significantly suppressed pyroptosis induced by OGD/R. Eth-D2 and YO-PRO-1 are membrane-impermeable dyes. YO-PRO-1, with a lower molecular weight, can penetrate the pores formed during pyroptosis, but Eth-D1, as it contains a higher molecular weight, cannot. Following staining, cells exhibiting green fluorescence are classified as pyroptotic, whereas cells showing both red and green fluorescence indicate cell death through alternative pathways. We performed Eth-D2 and YO-PRO-1 staining on BV2 cells treated with OGD/R and TA treatment, using 0.1% Triton as a control. As illustrated in Fig. 4N, O, the proportion of green-positive cells significantly increased in OGD/R cells, while the OGD/R + TA group showed a marked reduction in green-positive cells, indicating that TA effectively inhibited OGD/R-induced pyroptosis in BV2 cells.

### TA induced mitophagy via activation of PINK1/Parkin signaling pathway

**Fig. 5:**
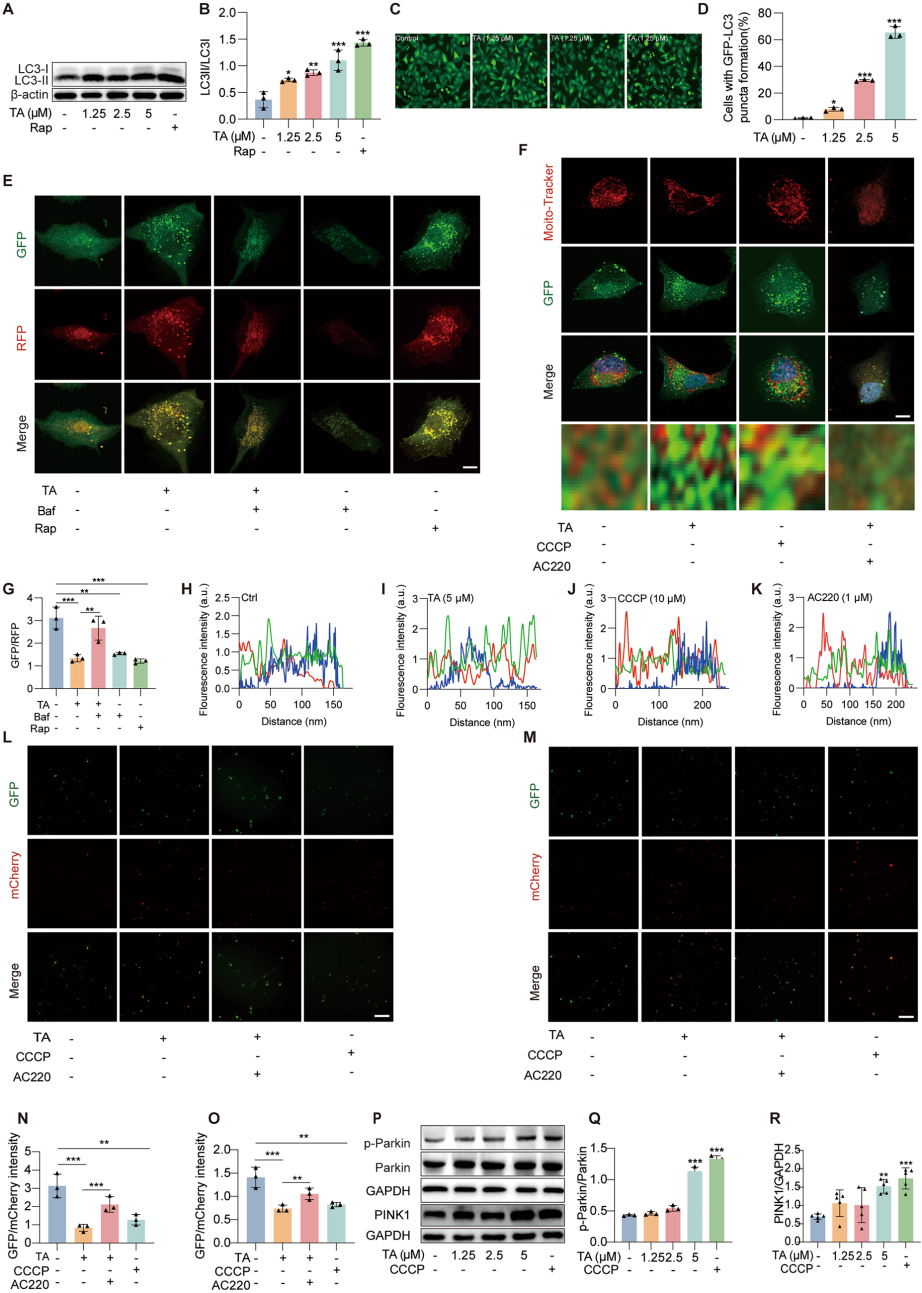
TA activated mitophagy by PINK1/Parkin signaling pathway. **A** The Western blotting detection of the protein expressions of LC3I and LC3II in HT22 cells treated with TA and Rap (20 μM). **B** The bar chart presents the rate of LC3II/LC3I, *p < 0.05, *p < 0.01 and ***p < 0.01 versus the control group, n = 3. **C** Representative images of stable GFP-LC3 U87 cells treated with or without TA, Magnification: 20 × , scale bar: 100 μm. **D** The bar chart presents the rate of cells with GFP-LC3 puncta formation in stable GFP-LC3 U87 cells, *p < 0.05 and ***p < 0.001 versus control group. **E** Representative images of stable stable RFP-GFP-LC3 U87 cells treated with or without TA, Baf (100 nM) or Rap (10 μM), Magnification: × 64, scale bar: 5 μm. **F** Representative images of the co-localization of GFP-LC3 and MitoTracker in stable GFP-LC3 U87 cells with or without TA, CCCP or TA+AC220. **G** The bar chart indicates the rate of GFP/RFP, **p < 0.05 and ***p < 0.001. **H**–**K** Line scan analysis showing the co-localization intensity (a.u.) of GFP-LC3 and MitoTracker in stable GFP-LC3 U87 cells under different treatments. **L** Representative images of HT22 cells transiently transfecting Mito-QC plasmid and treated with or without TA, CCCP or TA+AC220. **M** Representative images of BV2 cells transiently transfecting Mito-QC and treated with or without TA, CCCP or TA+AC220. **N** The bar chart indicates the rate of RFP/GFP in HT22 ransiently transfecting Mito-QC cells. **p < 0.01 and ***p < 0.001 versus the control group, n = 3. **O** The bar chart indicates the rate of RFP/GFP in BV2 ransiently transfecting Mito-QC cells. **p <0.01 and ***p < 0.001 versus the control group, n = 3. **P** Western blot detection of Parkin, p-Parkin, and PINK1 protein expression in HT22 cells treated with or without TA. **Q**, **R** The bar chart presents the rate of p-Parkin/Parkin (n = 3), PINK1/GAPDH (n = 5). **p < 0.01 and ***p < 0.001 versus the control group

Our previous research has shown that TA is the most potent autophagy activator from PCP [26]. Mitophagy is an important target for the intervention of CI/RI. Here, we reconfirmed the autophagy activation role of TA. As demonstrated in the Fig. 5A, B, Western blot analysis indicated that TA promoted an increase in the rate of LC3II in HT22 cells, and TA significantly activated autophagy in stable GFP-LC3 U87 cells (Fig. 5C, D). To determine whether TA can activate mitophagy, the mitochondrial fluorescent tracking dye Mito-Tracker along with Hoechst staining was employed. In stable RFP-GFP-LC3 U87 cells intervened by TA or autophagy agonist Rap, there were significant red and green fluorescent spots, and the relative ratio of GFP/RFP increased significantly, in the autophagy inhibitor Bafilomycin (Baf) group, the ratio of GFP/RFP decreased significantly. The above results suggest that TA induced autophagy (Fig. 5E, G). Additionally, using MitoTracker staing in stable GFP-LC3 U87 cells, we found that TA enhances the co-localization of LC3 and mitochondria. These findings collectively suggest that TA activates mitophagy (Fig. 5F, H–K). Furtherly, mCherry-GFP-FIS1-293 T cells were treated with TA and mitophagy inducer chlorophenylhydrazone (CCCP). Under normal conditions, mitochondria exhibit green fluorescence, during mitophagy, the autophagosomes fuse with lysosomes, creating an acidic environment that diminishes or eliminates the green fluorescence while the red fluorescence remains stable or increasing. As shown in Fig. S8, the GFP/RFP ratio decreased in the TA or CCCP group, whereas in the group treated with both TA and AC220, a mitophagy inhibitor, the GFP/RFP ratio increased, these results clearly indicated that TA activated mitophagy. Furthermore, in HT22 and BV2 cells, transient transfection of Mito-QC plasmids followed by treatment with or without TA, CCCP, or TA + AC220 revealed that TA activates mitophagy in both cell lines (Fig. 5L-O). Previous studies have established that mitophagy is primarily regulated by the PINK1/Parkin pathway [27]. In this study, Western blot analysis demonstrated that both TA and CCCP enhanced the expression of p-Parkin and PINK1 proteins (Fig. 5P–R).

### TA inhibited OGD/R-induced apoptosis by activating mitophagy in HT22 cells

**Fig. 6:**
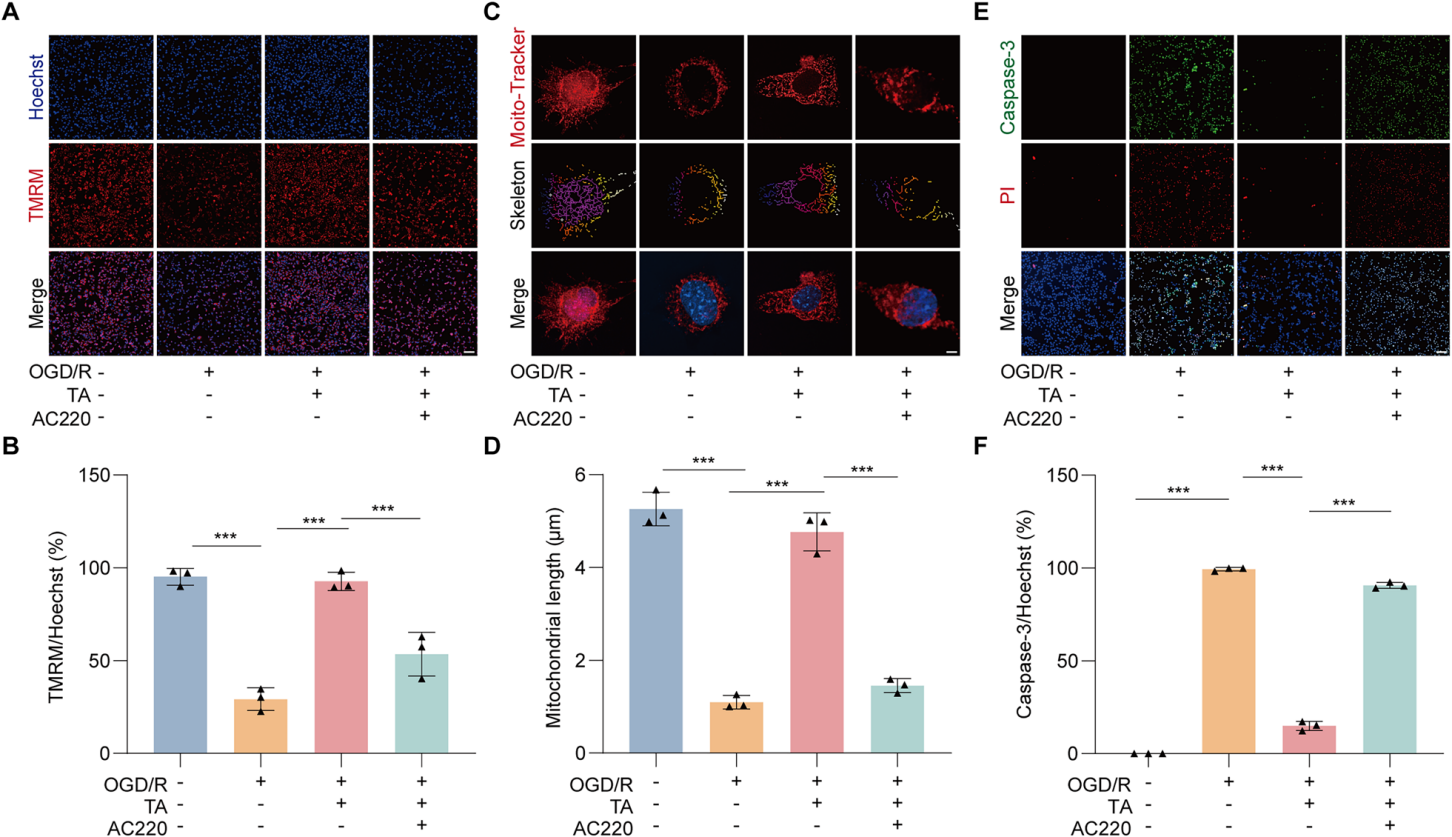
TA inhibited OGD/R-induced apoptosis by activating mitophagy in HT22 cells. **A** HT22 cells were subjected to OGD/R and treated with either TA or TA combined with AC220. Cells were then stained with Hoechst and TMRM to assess MMP changes. Fluorescence images were acquired at a magnification of × 10; scale bar represents 200 μm. **B** The bar chart indicates the rate of TMRM/Hochest (%) in HT22 cells. ***p < 0.001 versus the the corresponding group, n = 3. **C** Representative images of MitoTracker staing in HT22 cells subjected to OGD/R and treated with either TA or TA combined with AC220. Magnification: 64 × , scale bar: 5 μm. **D** The bar chart shows the mitochondrial length of HT22 cells in different groups. ***p < 0.001 versus the the corresponding group, n = 3. **E** Representative images of Caspase3 and PI staining in HT22 cells subjected to OGD/R and treated with either TA or TA combined with AC220. Magnification: 10 × scale bar: 100 μm. **F** The bar chart indicates the rate of Caspase3/Hoechst of HT22 cells in different groups, ***p < 0.001 versus the corresponding group, n = 3

We further explored whether TA could protect HT22 cells from apoptosis induced by OGD/R injury through the activation of mitophagy. We initially utilized TMRM, a dye that selectively accumulates in mitochondria, driven by the MMP, to assess changes in MMP. As illustrated in Fig. 6A, B, following OGD/R, there was a notable reduction in orange-red fluorescence indicating the TMRM accumulation and Hoechst blue fluorescence, suggesting a decline in MMP. Conversely, treatment with TA resulted in an enhanced orange-red fluorescence signal, while the mitophagy inhibitor AC220 was able to reverse this effect. Moreover, Mito-Tracker result analysis demonstrated that TA significantly mitigated OGD/R-induced mitochondrial damage, with AC220 similarly reversing this protective effect (Fig. 6C, D). Subsequently, we conducted Caspase-3 fluorescence staining, which revealed an increase in the number of Caspase-3 positive cell in the OGD/R group compared to the control group. In contrast, TA treatment effectively reduced the number of Caspase-3 positive signals (Fig. 6E, F). Notably, when autophagy was inhibited with AC220, the previously reduced Caspase-3 levels increased again. This indicates that TA can inhibit OGD/R-induced apoptosis in HT22 cells through the activation of mitophagy.

### TA inhibited OGD/R-induced pyroptosis by activating mitophagy in BV2 cells

**Fig. 7:**
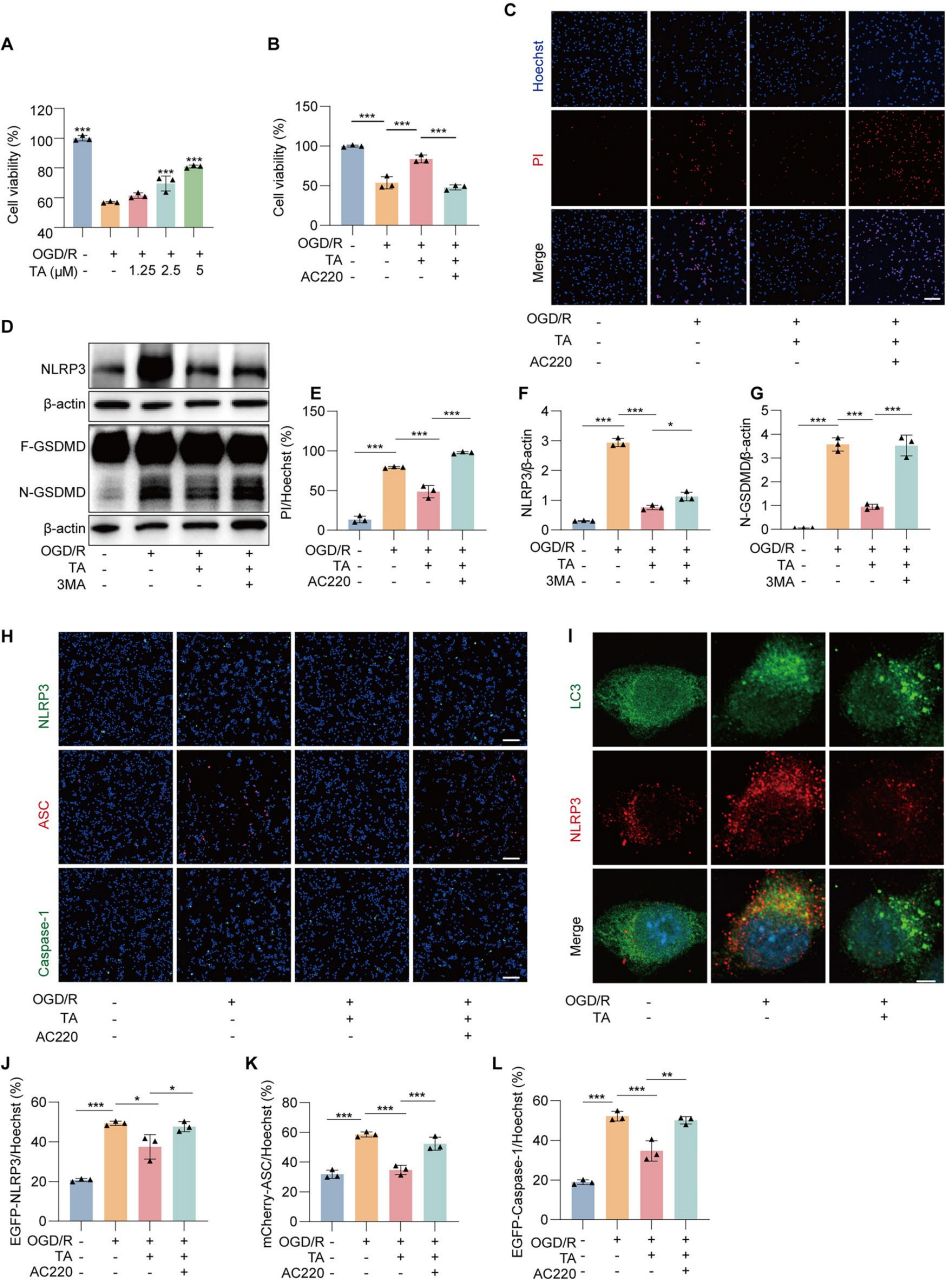
TA inhibited OGD/R-induced pyroptosis by activating mitophagy in BV2 cells. **A** BV2 cells were subjected to OGD/R and treated with or wthout TA, then cell viability were detected by MTT.***p < 0.001 versus OGD/R alone group, n = 3. **B** BV2 cells were subjected to OGD/R and treated either TA or TA combined with mitophagy inhibitor AC220, then cell viability was detected by MTT. **p < 0.01 and ***p < 0.001 versus the corresponding group, n = 3. **C** Representative images of Hoechst and PI staining in BV2 cells subjected to OGD/R and treated with either TA or TA combined with AC220. Magnification: × 10; scale bar: 100 μm. **D** The Western blotting detection of the protein expressions of NLRP3 and GSDMD in BV2 cells subjected to OGD/R and treated with either TA or TA combined with atophagy inhibitor 3MA. **E** The bar chart indicates the rate of PI/Hoechst in BV2 cells. ***p < 0.001, n = 3. **F**, **G** The bar chart presents the ratios of NLRP3/β-actin, N-GSDMD/β-actin. * *p* < 0.05 and ***p < 0.001versus the corresponding group, n = 3. **H** Representative images of BV2 cells transfected with EGFP-NLRP3, EGFP-Caspase-1 or mCherry-ASC plasmid and subjected to OGD/R and treated with either TA or TA combined with AC220. Magnification: × 10, scale bar: 100 μm. **I** BV2 cells were subjected to OGD/R and treated with or without TA, immunofluorescence staining was used to observe the co-localization of LC3 and NLRP3, and fluorescence images were collected. Magnification: × 64, scale bar: 5 μm. **J**–**L** The bar chart presents the ratios of EGFP-NLRP3/Hoechst, EGFP-Caspase-1/Hoechst or mCherry-ASC/Hoechst in BV2 cells of different groups. *p < 0.05, **p < 0.01 and ***p < 0.001, n = 3

To verify the protective effect of TA on BV2 cells against OGD/R injury by activating mitophagy, firstly, BV2 cell viability was detected by MTT, the results showed that TA could improve the survival rate of cells induced by OGD/R (Fig. 7A), and both the autophagy inhibitor 3-MA (Fig. S9) and mitophagy inhibitor AC220 could reverse the effect of the recovery of BV2 cell viability (Fig. 7B). Hoechst/PI staining, demonstrated that TA reduced the rate of Hoechst/PI in BV2 cells induced by OGD/R and the effect was reversed by AC220 indicating that TA can protect BV2 cells from OGD/R injury by activating mitophagy (Fig. 7C, E). To investigate whether TA activates autophagy and mitophagy to suppress OGD/R-induced pyroptosis, the expression levels of NLRP3 and GSDMD were detected in BV2 cells treated with TA in combination with the 3-MA after OGD/R induction. Figure 7D, F, G indicated that OGD/R significantly increased the expression of NLRP3 and N-GSDMD, which was notably reduced by TA and reversed by 3-MA, suggesting that TA inhibited OGD/R-induced pyroptosis via autophagy activation. Moreover, to confirm whether TA inhibits OGD/R-induced pyroptosis in BV2 cells by activating mitophagy, we transiently plasmid EGFP-N1-NLRP3, mCherry-C1-ASC, and EGFP-N1-caspase-1 into BV2 cells, following OGD/R induction, cells were treated with TA or TA+AC220, the changes in fluorescence intensity were observed using a high-content imaging system (Fig. 7H), further revealed that TA effectively reduced the green fluorescence intensity of NLRP3 and caspase-1, as well as the red fluorescence of ASC (Fig. 7J–L), all of which were reversed by mitophagy inhibitor AC220. Furthermore, as Fig. 7I, NLRP3 and LC3 co-localization staining showed TA can promote the expression of LC3 in BV2 cells under OGD/R conditions, while reducing the expression of NLRP3 and decreasing the co-localization of the two. This suggests that TA can inhibit OGD/R- induced pyroptosis through the activation of mitophagy.

### TA ameliorated neurological injury in MCAO/R rats by activating mitophagy via the PINK1/Parkin signaling pathways

**Fig. 8:**
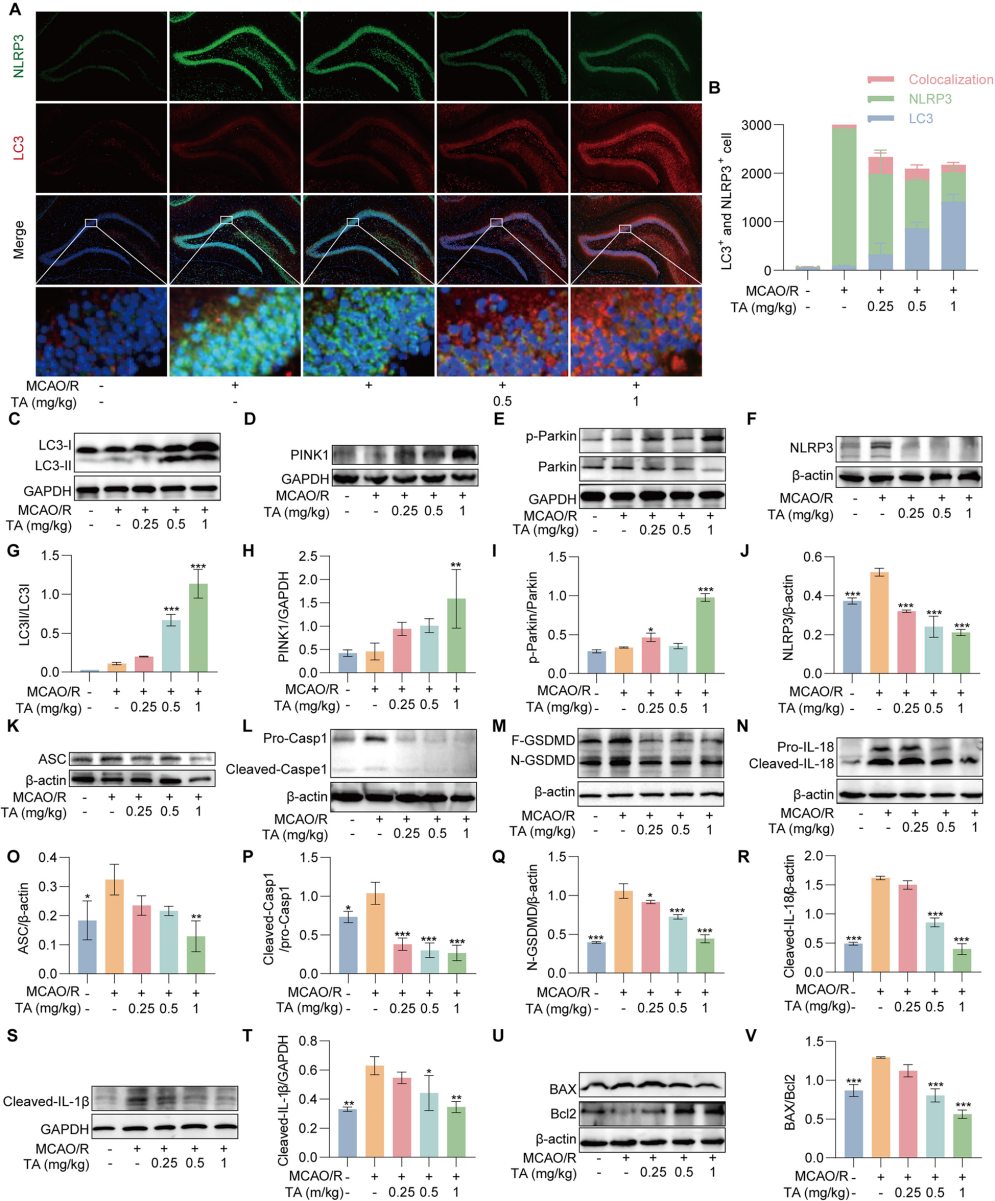
TA ameliorated neurological injury in MCAO/R Rats. **A** Representative immunofluorescence staining images of LC3 and NLRP3 in hippocampus of the brain in MCAO/R rats, × 10, scale bar: 100 μm. **B** The bar chart presents the counts of LC3^+^ cells, NLRP3^+^/cells, and LC3^+^/NLRP3 ^+^ co-localized cells. **C**–**F** The Western blotting detection of the protein expressions of LC3, PINK1, p-Parkin and NLRP3 in rat brain tissues across experimental groups. **G**–**J** The bar chart presents the ratios of LC3-II/I, PINK1/β-actin, p-Parkin/ Parkin, NLRP3/β-actin. *p < 0.05, **p < 0.01 and ***p < 0.001versus the alone MCAO/R group, n = 3. **K**–**N** The Western blotting detection of the protein expressions of ASC, Caspase-1, IL-18 in rat brain tissues across experimental groups. **O**–**R** The bar chart presents the ratios of ASC/β-actin, Cleaved-Caspase-1/Pro- Caspase-1, Cleaved-IL-18/β-actin. *p < 0.05, **p < 0.01 and ***p < 0.001versus the alone MCAO/R group, n = 3. **S** The Western blotting detection of the protein expression of IL-1β. **T** The bar chart presents the rate of Cleaved-IL-18/GAPDH. *p < 0.05 and **p < 0.01 versus the alone MCAO/R group, n = 3. **S** The Western blotting detection of the protein expression of IL-1β. **T** The bar chart presents the rate of Cleaved-IL-1β/GAPDH. *p < 0.05 and **p < 0.01 versus the alone MCAO/R group, n = 3. **U** The Western blotting detection of the protein expression of BAX and Bcl2. **V** The bar chart presents the rate of BAX/Bcl2. ***p < 0.001 versus the alone MCAO/R group, n = 3

We employed immunofluorescence to assess the expression and co-localization of LC3 and NLRP3 in the brain tissue of MCAO/R rats. As illustrated in Fig. 8A, B, in hippocampus of the brain, treatment with TA markedly enhanced the expression of LC3 while concurrently reducing the expression of NLRP3, along with decreasing the co-localization of these proteins. Furthermore, as Fig. 8C-T, Western blot analysis demonstrated that various concentrations of TA led to an increase in the levels of LC3, PINK1, and p-Parkin in the brain tissue of MCAO/R rats. Notably, TA treatment reduced the expression of NLRP3, ASC, Cleaved-caspase-1, N-GSDMD, Cleaved-IL-1β and Cleaved-18, also resulted in a decrease in the BAX/Bcl-2 ratio (Fig. 8U-V). These findings indicated that TA activates mitophagy through the PINK1/Parkin signaling pathway, thereby improving neurological function in MCAO/R rats.

## Discussion

IS presents a significant challenge to public health worldwide with complex pathophysiological processes underlying CI/RI [48]. These processes involve multiple mechanisms, including calcium overload, inflammatory responses, excitotoxicity from amino acids, disruptions in energy metabolism, and oxidative stress [30]. Mitochondria, known as the "powerhouses" of the cell, play a critical role in these pathological mechanisms [29]. During the ischemia/reperfusion events, mitochondrial function is severely compromised including mitochondrial DNA damage, excessive production of ROS, and imbalances in mitochondrial dynamics, such as fission and fusion [30]. These changes contribute to various forms of programmed cell death, including apoptosis, pyroptosis, necroptosis, and ferroptosis [31]. Mitophagy is a selective process that targets and degrades dysfunctional mitochondria, thereby maintaining the integrity of the mitochondrial network and restoring cellular homeostasis [32]. Consequently, the activation of mitophagy emerges as a promising therapeutic strategy to counteract programmed cell death and mitigate secondary brain injury associated with severe ischemia/reperfusion [33].

CI/RI induces the production and accumulation of ROS, leading to oxidative stress damage in mitochondria [34]. Accumulating evidence demonstrates that excessive ROS can modulate the activity of Nrf2, a pivotal transcription factor involved in the antioxidant response, and regulates the expression of downstream antioxidant genes, including heme HO-1, NQO1, and GCLC, thereby maintaining cellular redox homeostasis and protecting cells from oxidative damage [35]. In the present study, we validated that OGD/R significantly elevated ROS levels in HT22 cells, while TA effectively inhibited ROS release. Additionally, TA treatment improved MMP and enhanced mitochondrial activity. Moreover, in TA-treated cells, we observed a significant increase in the levels of antioxidant-related proteins, including Nrf2, HO-1, NQO1, and GCLC. These results suggest that TA may serve as a potential therapeutic agent for protecting against CI/RI-induced mitochondrial oxidative damage.

Apoptosis is a form of programmed cell death that allows cells to systematically terminate their own life in response to specific physiological or pathological conditions [36]. This mechanism is distinct from necrosis, which involves the death of aging, dysfunctional, or damaged cells [37]. Recent research has underscored the critical role of apoptosis in the pathological processes associated with CI/RI [38]. ROS leads to mitochondrial membrane damage and subsequent release of cytochrome c. This event is accompanied by a decrease in the expression of anti-apoptotic proteins such as Bcl-2 and Bcl-XL, while pro-apoptotic factors, including caspase-3 and Caspase-9, become activated, thereby triggering the apoptotic pathway [39]. Among them, Caspase-3 and Caspase-7, as the main executor caspases, synergistically cleave multiple cellular substrates, ultimately leading to typical apoptotic changes in cell morphology and function. In this study, TA treatment significantly reduced the cell apoptosis rate and reversed the expression levels of apoptosis-related proteins including Caspase-9, Caspase-3, and Caspase-7, suggesting that TA can effectively inhibit the mitochondrial pathway-mediated apoptosis response induced by OGD/R. Furthermore, after HT22 cells were cultured by using the supernatant collected from BV2, TA significantly reduced the increase in Caspase-3 fluorescence resulting from OGD/R, providing strong evidence of its inhibitory effect on HT22 cell apoptosis induced by inflammatory cytokines released by BV2 cells. In addition, ROS also leads to the activation of NLRP3, Pro-Caspase-1 is recruited by ASC to form a filamentous protein complex called the ASC speck, and Pro-Caspase-1 is cleaved to generate active caspase-1, which further cleaves pro-IL-1β and pro-IL-18 to produce IL-1β and IL-18 [40]. The cleaved Caspase-1 can also specifically cleave GSDMD to release the N-terminal pore-forming domain, ultimately leading to pyroptosis [41]. By using Yo-Pro-1/EthD-1 dyes, we observed the effect of TA on the positivity rate of pyroptosis induced by OGD/R in BV2 cells, decreasing the cell membrane permeability through GSDMD, and effectively improved OGD/R-induced pyroptosis in BV2 cells by detecting pyroptosis-related proteins [42].

Mitophagy, the selective degradation of damaged mitochondria, plays a vital role in maintaining mitochondrial homeostasis and reducing oxygen consumption during cellular stress. Neurons experience ischemia and hypoxia, which can lead to early mitochondrial dysfunction in IS. The removal of damaged mitochondria through autophagy is essential for protecting neurons from apoptosis and necrosis [43]. When cells endure ischemic and hypoxic damage within tolerable limits, they can activate mitophagy to facilitate self-repair and mitigate injury [45]. Disruption of this process may present a viable therapeutic target for IS. Moderate activation of mitophagy can provide protective effects on hypoxic neurons, alleviating CI/RI [43]. Conversely, insufficient autophagic function may lead to inadequate clearance of damaged mitochondria, while excessive activation could result in mitochondrial overload, disrupting cellular homeostasis and exacerbating apoptosis or necrosis, ultimately worsening ischemic or ischemia/reperfusion injuries [44]. PINK1 is a key regulator of mitophagy. Mitochondria that exhibit compromised structure and reduced membrane potential activate PINK1, which recruits Parkin, an E3 ubiquitin ligase, to the mitochondrial surface, initiating the autophagic process [45]. Numerous studies have shown that the PINK1/Parkin-mediated autophagy pathway is integral to the pathogenesis of various disorders, including stroke, neurodegenerative diseases, and multiple sclerosis [46]. Activation of the PINK1/Parkin mitophagy signaling pathway can clear oxidatively damaged mitochondria, thereby maintaining normal mitochondrial homeostasis and reducing cell death [10]. Our previous research indicated that TA possesses autophagy-activating properties [26]. Therefore, we aimed to investigate whether TA provides protection against brain CI/RI through the activation of autophagy and mitophagy. In this study, autophagy and mitophagy inhibitors and agonists were applied to demonstrate the effects of TA on activating autophagy and mitophagy, and proved TA activates mitophagy via the PINK1/Parkin pathway. Previous studies demonstrated that TA activates autophagy via Ca^2^⁺/AMPK-dependent and mTOR-independent pathways [26], our research further clarifies TA's role and the specific pathway involved in activating mitophagy by PINK1/Parkin mitophagy signaling pathway in the CI/RI cell model simulated by OGD/R. Subsequently, we proved TA could inhibit OGD/R-induced apoptosis and pyroptosis through the activation of mitophagy.

The middle cerebral artery is a common site for stroke, making the MCAO/R animal model widely recognized as the standard model for studying CI/RI due to its pathogenic mechanisms that closely resemble those observed in humans [46]. At first, we established an MCAO/R model in SD rats and administered TA treatment, proved that TA significantly improved cognitive function and reduced infarct area, and immunofluorescence analysis revealed that TA inhibited the proliferation of Iba-1 and GFAP while increasing the number of NeuN-positive neurons. Compared with the positive control drug NMDP (10 mg/kg), high-dose (1 mg/kg) of TA demonstrated superior efficacy in improving neurobehavioral outcomes, reducing TTC-stained infarct volume, downregulating GFAP and IBA1 expression, and upregulating NeuN expression, As a naturally derived compound, TA exhibits robust neuroprotective effects, underscoring its potential as a therapeutic candidate for IS. After completing a series of cell tests, in MCAO/R rats, it was reproved that TA treatment resulted in a decreased BAX/Bcl2 ratio and reduced expression of pro-apoptotic proteins. Furthermore, co-localization studies of LC3 and NLRP3 demonstrated that TA significantly enhanced the positivity rate of LC3, decreased NLRP3 expression, and reduced the co-localization of the two proteins. Protein assays conducted on brain tissue from MCAO/R rats confirmed that TA activates mitophagy in neurons through the PINK1/Parkin signaling pathway.

Based on this study, Future studies should explore the specific molecular target of TA in activating the PINK1/Parkin pathway, determine whether this pathway activation also underlies the inhibition of other forms of cell death (such as necroptosis), and investigate if sustained pathway activation is critical for long-term neurological functional recovery in CI/RI or IS.

## Conclusion

Our comprehensive investigation, spanning from cellular models to animal studies, has convincingly demonstrated that TA extracted from PCP exerts a potent neuroprotective effect against CI/RI by activating mitophagy, this process effectively mitigates neuronal apoptosis and pyroptosis. Our findings significantly broaden the traditional scope of TA's applications, transcending its established roles in liver protection and function enhancement to encompass a novel therapeutic avenue for IS. Nevertheless, we must recognize that significant uncertainty remains regarding the complex molecular mechanisms and interactions underlying the neuroprotective effects of PCP and its extracts. To fully explore the therapeutic potential of PCP in treating neurological diseases, further in-depth research is essential.

## Supplementary Information

Supplementary material 1.

## Data Availability

The datasets used and/or analyzed among the current study are available from the corresponding author on reasonable request.

## Abbreviations

AD: Alzheimer's disease
Baf: Bafilomycin
CI/RI: Cerebral ischemia/reperfusion injury
CCA: Common carotid artery
CCCP: Carbonyl cyanide 3-chlorophenylhydrazone
DHE: Dihydroethidium
ECA: External carotid artery
GCLC: Glutamate-cysteine ligase catalytic subunit
GSDMD: Gasdermin D
HO-1: Heme oxygenase-1
IS: Ischemic stroke
ICA: Internal carotid artery
MMP: Mitochondrial membrane potential
MCA: Middle cerebral artery
NMDP: Nimodipine
Nrf2: Nuclear factor erythroid 2-related factor2
NQO1: NADPH quinone oxidoreductase1
PCP: *Penthorum chinense* Pursh
p-Parkin: Phosphorylated Parkin
PINK1: PTEN-induced putative kinase 1
Rap: Rapamycin
ROS: Reactive oxygen species
SD: Sprague–Dawley
TA: Thonningianin A
TCMs: Traditional Chinese medicines
TMRM: Tetramethylrhodamine Methyl Ester
