# Supplementary material for: Thonningianin A derived from Penthorum chinense Pursh alleviates cerebral ischemia/reperfusion-mediated apoptosis and pyroptosis through the activation of PINK1/Parkin-dependent mitophagy
Source: Chin Med. 2026 Jan 16;21:40. doi: 10.1186/s13020-025-01247-2 (PMC12809999; doi:10.1186/s13020-025-01247-2)
Supplement: Supplementary file 1 — Supplementary material 1. [file 13020_2025_1247_MOESM1_ESM.docx]

**Supplementary materials**

**Thonningianin A derived from *Penthorum chinense* Pursh alleviates cerebral ischemia/reperfusion-mediated apoptosis and pyroptosis through the activation of PINK1/Parkin-dependent mitophagy
Qianfang Yao^1#^, Guishan Hu^1^****^#^, Can Yin****^1#^, Anguo Wu^1^, Guangqiang Hu^2^, Dalian Qin^1^, Xiaogang Zhou^1^, Betty Yuen-Kwan Law^3^, Xi Du^4^, Li Chen^5^, Jianqiao Li^1^, Hong Lin^2^, long xin^6^, Jianming Wu****^1*^, Lu Yu^1*^**

^1^Sichuan Key Medical Laboratory of New Drug Discovery and Drugability Evaluation, School of Pharmacy, School of Basic Medical Sciences, Southwest Medical University Luzhou 646000, China

^2^Department of Anatomy, School of Basic Medical Sciences, Southwest Medical University, Luzhou 646000, China

^3^State Key Laboratory of Quality Research in Chinese Medicine, Macau University of Science and Technology, Taipa, Macau

^4^Department of Chemistry, School of Basic Medical Sciences, Southwest Medical University, Luzhou 646000, China

^5^Department of Neurology, The Affiliated Traditional Chinese Medicine Hospital of Southwest Medical University, Luzhou, 646000 Sichuan China.

^6^Clinical Medical College of Southwest Medical University, Luzhou, 646000 Sichuan China.

^*^Correspondence yulu863@swmu.edu.cn; jianmingwu@swmu.edu.cn


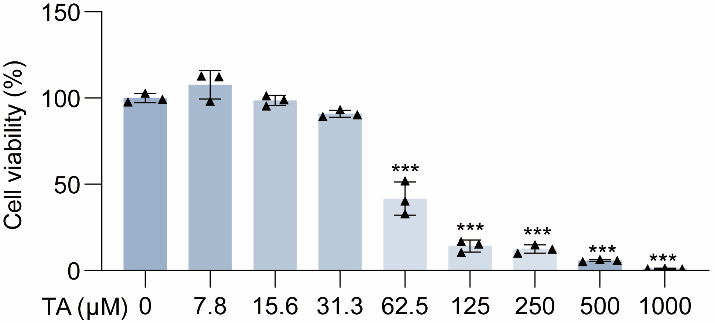


**Fig. S1** Cytotoxicity assay of TA on HT-22. HT22 cells were seeded in 96-well plates. After 24 h, TA was serially diluted from a highest concentration of 1000 µM and added into the cells. Following another 24 h incubation, MTT assay was performed to assess the cytotoxicity of TA on HT22 cells. The bar chart showing the cell viability. ****p* < 0.001 versus without TA group, n = 3.


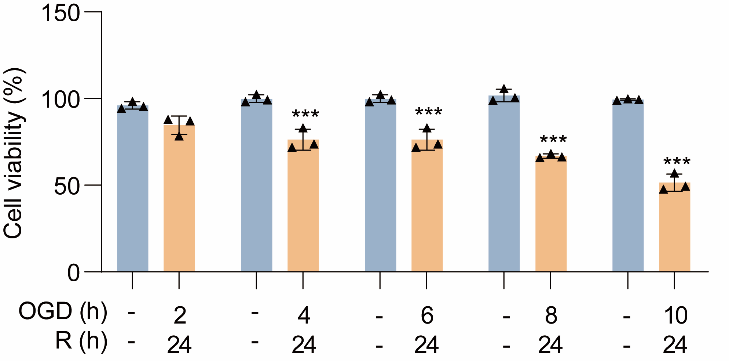


**Fig. S2** Time screening for OGD/R model construction in HT22 cells. Following OGD/R treatment at different time points, cell toxicity was assessed using the MTT to explore the optimal time point for establishing the OGD/R model. ****p* < 0.001 versus nomal control group, n = 3.


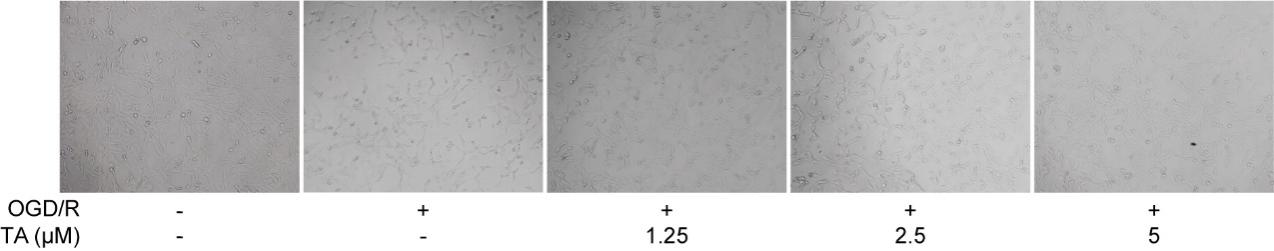


**Fig. S3** HT22 cell state following OGD/R treatment. Cells were subjected to OGD for 10 h, followed by 24 h of reperfusion, cell morphology was then examined under a microscope and images were captured. Magnification: ×10; Scale bar: 200 μm.


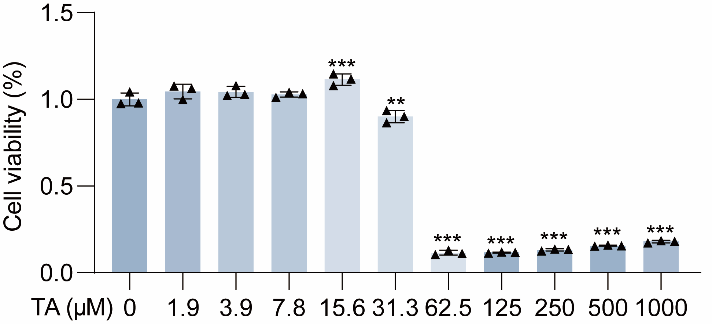


**Fig. S4** Cytotoxicity assay of TA on BV2. BV2 cells were planted in 96-well plates. After 24 h, TA was added into the cells from a highest concentration of 1000 µM to 1.9 µM. Following another 24 h incubation, MTT assay was performed to assess the cytotoxicity of TA in BV2 cells. The bar chart showing the cell viability, ***p* < 0.01 and ****p* < 0.001 versus without TA group, n = 3.


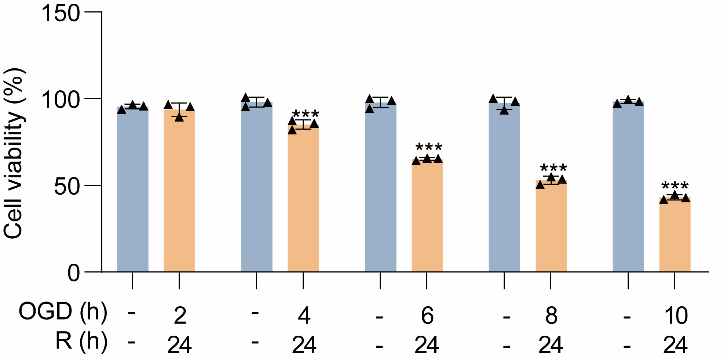


**Fig. S5** Time screening for OGD/R model construction in BV2 cells. Following OGD/R treatment administered at different time points, cell toxicity was assessed using the MTT assay to explore the optimal time point for establishing the OGD/R model.****p* < 0.001 versus nomal control group, n = 3.


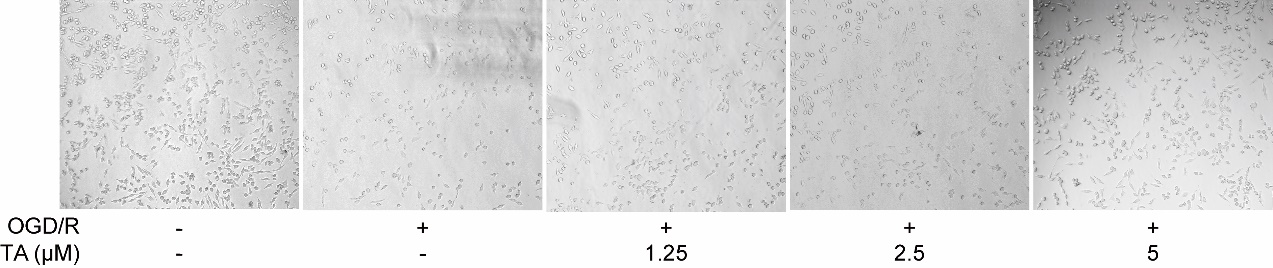


**Fig. S6** BV2 cell state following OGD/R treatment. Cells were subjected to OGD for 10 h, followed by 24 h of reperfusion, cell morphology was then examined under a microscope and images were captured. Magnification: ×10; Scale bar: 200 μm.

**
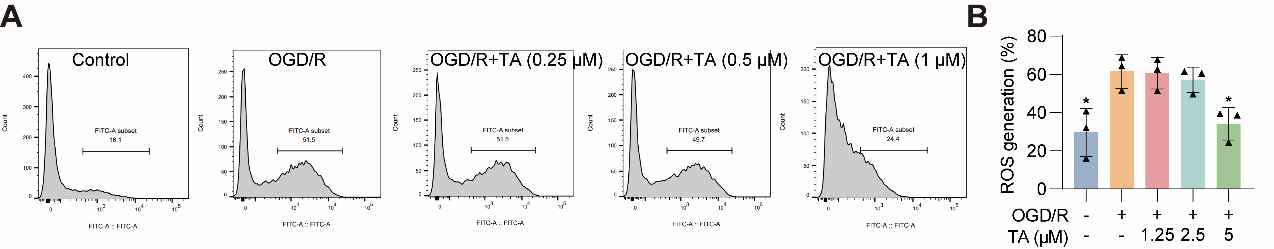
Fig. S7** TA significantly reduced ROS generation induced by OGD/R in HT22 cells. HT22 cells were subjected to OGD/R with or without TA treatment, cells were collected and centrifuged, DHE solution was added to cells and incubated in a 37 °C incubator for 20 min, then ROS generation were detected by flow cytometry. **p* < 0.05 versus alone OGD/R group, n = 3.


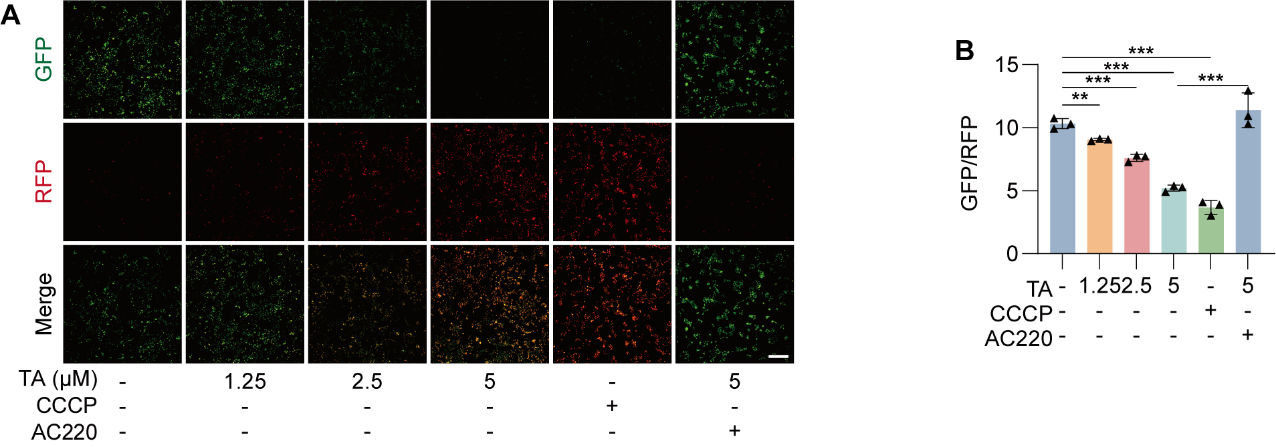


**Fig. S8** TA activates mitophagy in mCherry-GFP-FIS1-293T cells. After 24 hours of culture, the cells were exposed to various concentrations of TA (1.25 μM, 2.5 μM and 5 μM), CCCP (10 μM), or TA (5 μM ) + AC220 (10 μM) for 24 h and then imaged using a fluorescence microscope. Magnification: ×10 , scale bar: 200 μm. The bar chart showes the the rate of GFP/RFP. ***p* < 0.05 and ****p* < 0.001, n = 3..

**
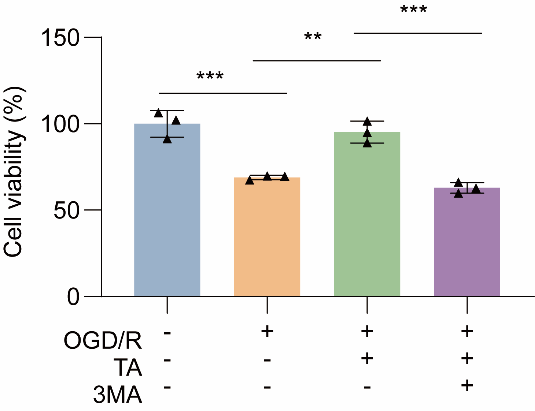
Fig.S9** TA inhibited OGD/R-induced BV2 cells death through activation of autophagy. BV2 cells were subjected to OGD/R and treated with TA (5 μM), 3MA (5 mM) or TA + 3MA. The cell viability were detected by using MTT. The bar chart indicates cell viability of BV2 cells. ***p* < 0.01 and ****p* < 0.001, n = 3.


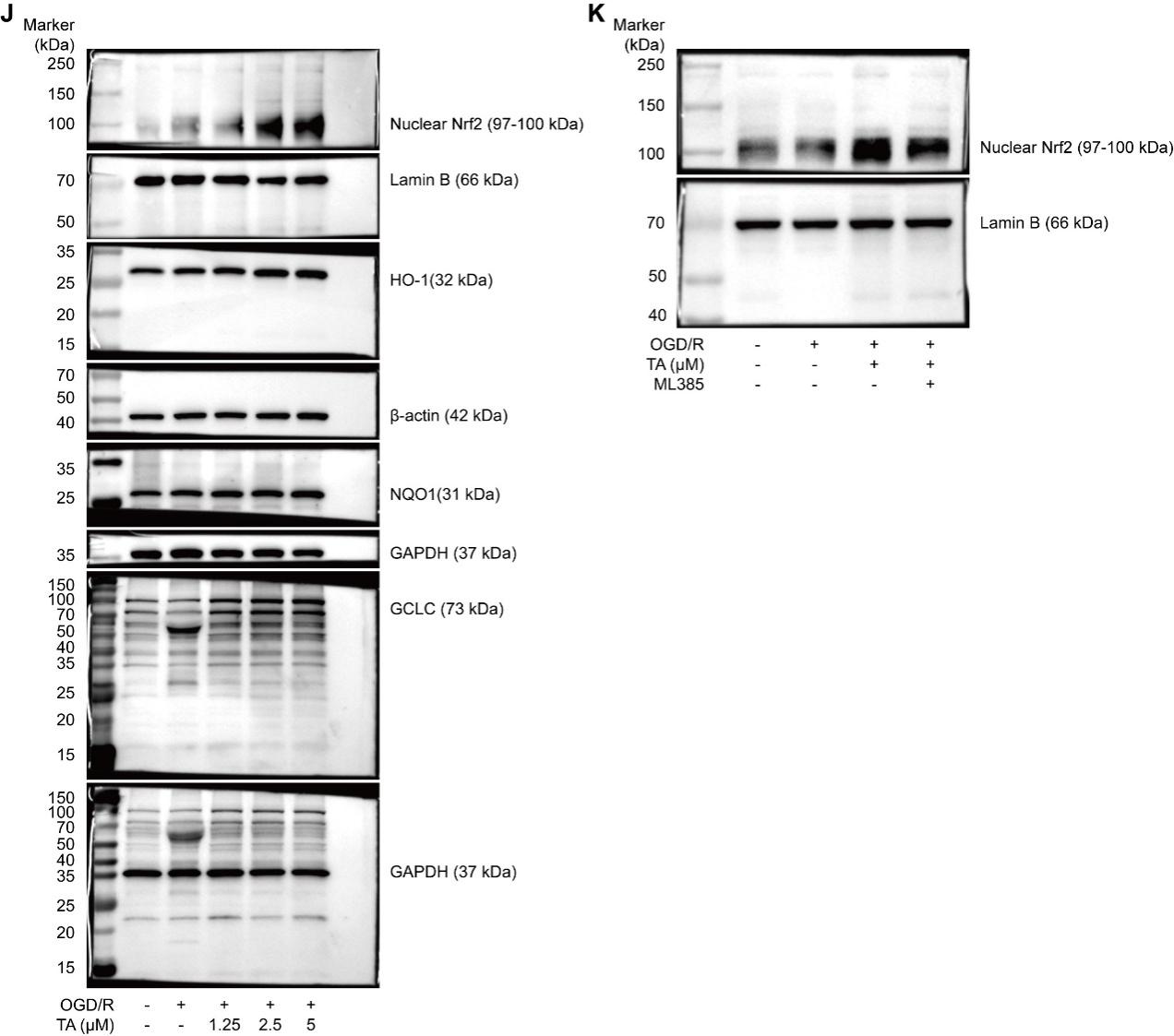


**Fig.S10** The original Western blot images of Fig. 2J, Fig. 2O


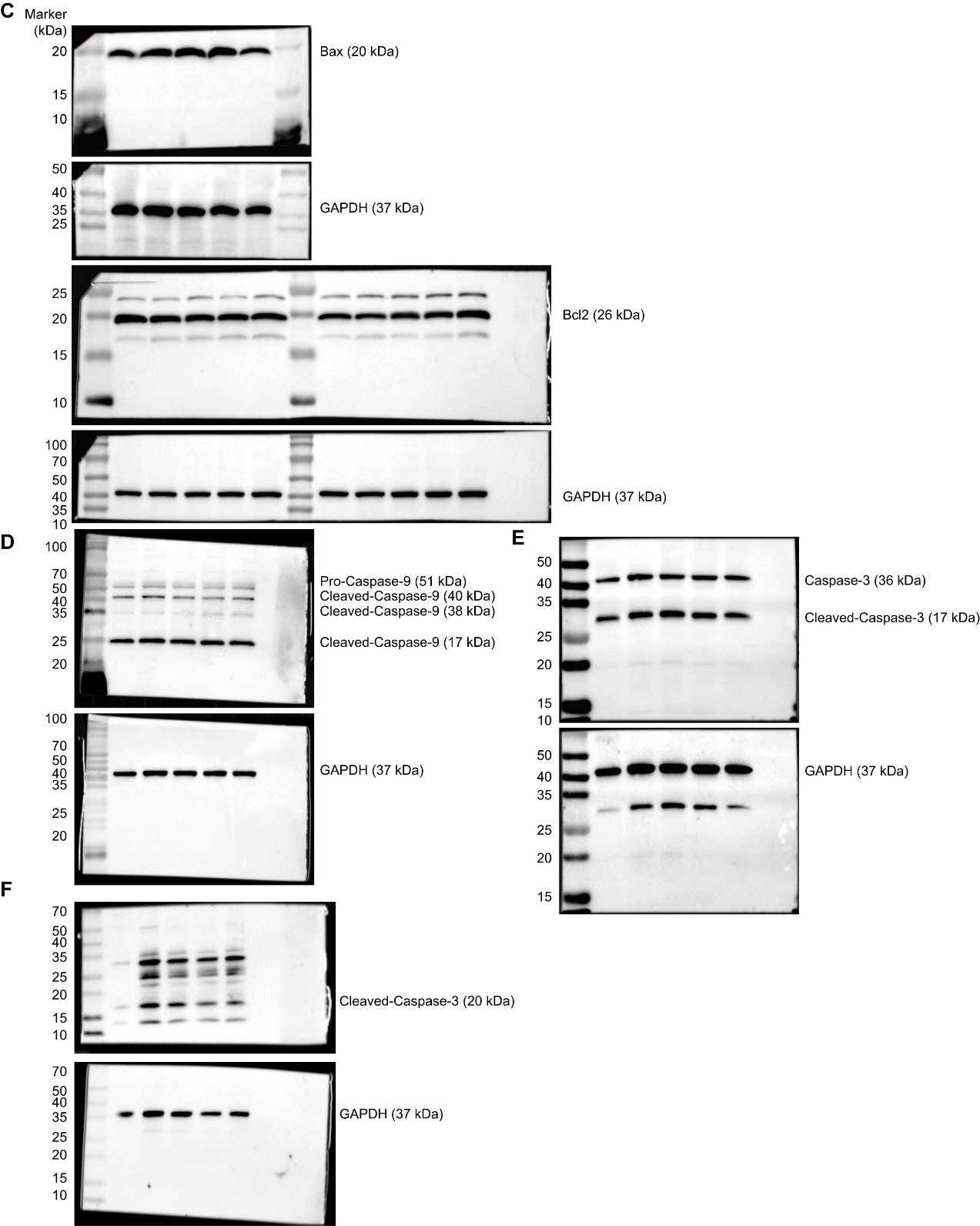


**Fig. S11** The original Western blot images of Fig.3C, D, E, F.


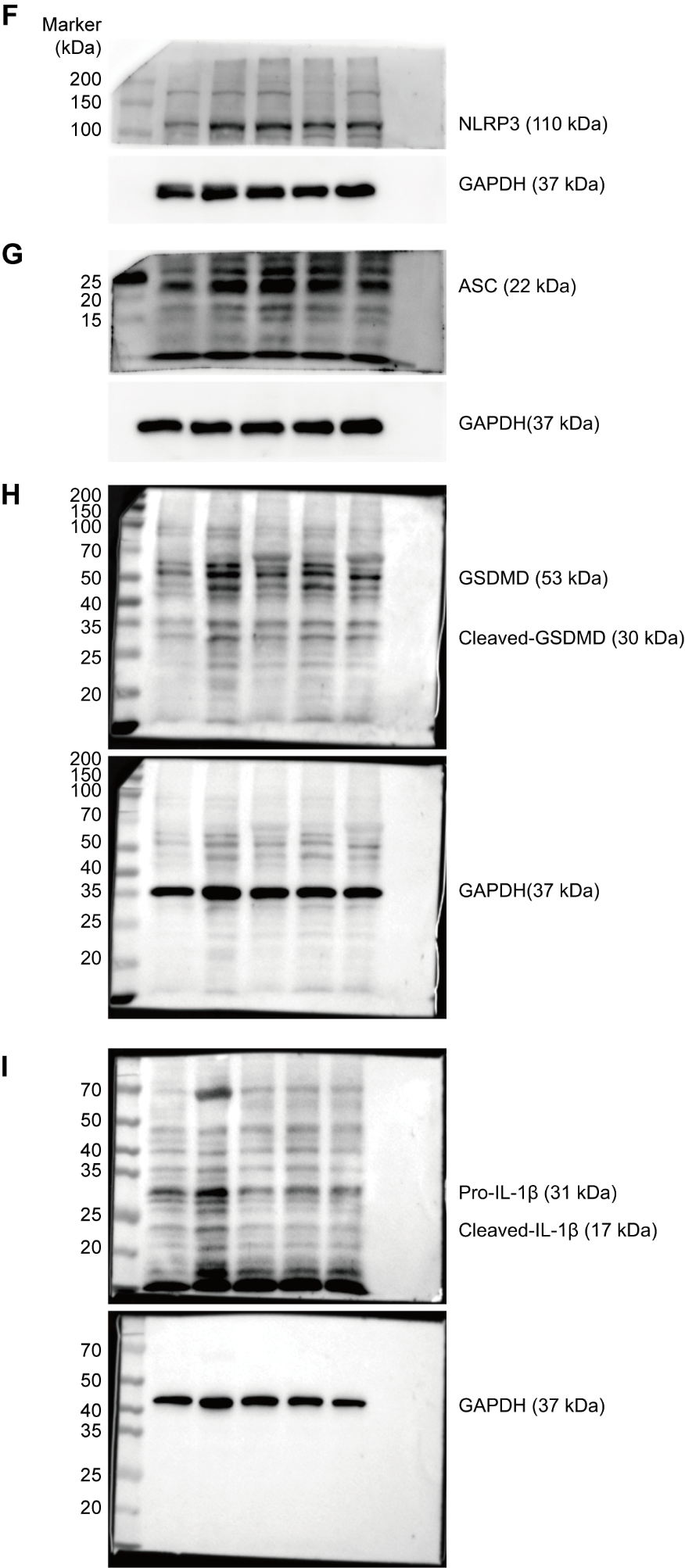


**Fig. S12** The original Western blot images of Fig.4F, G, H, I.


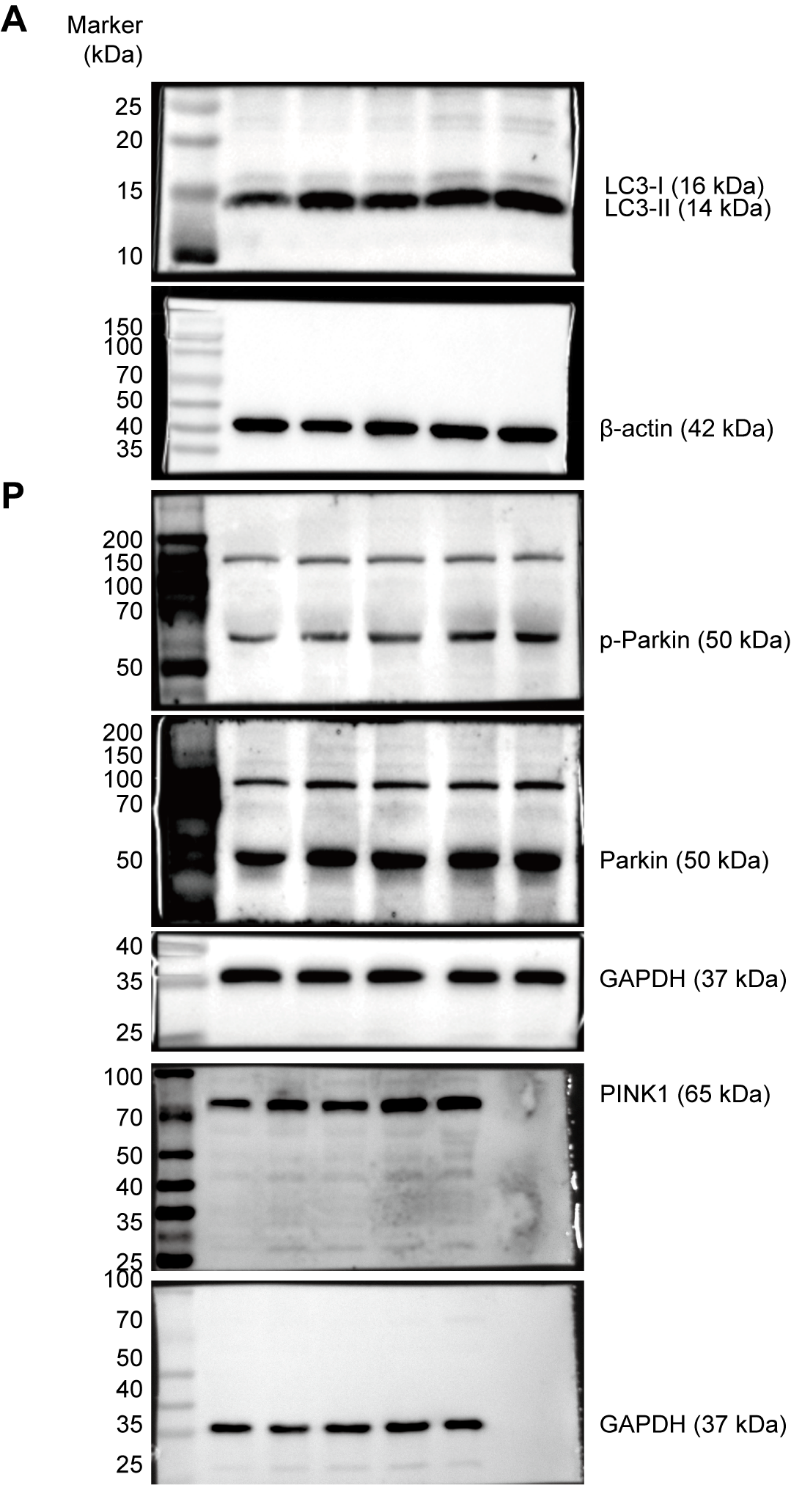


**Fig. S13** The original Western blot images of Fig.5A, G.


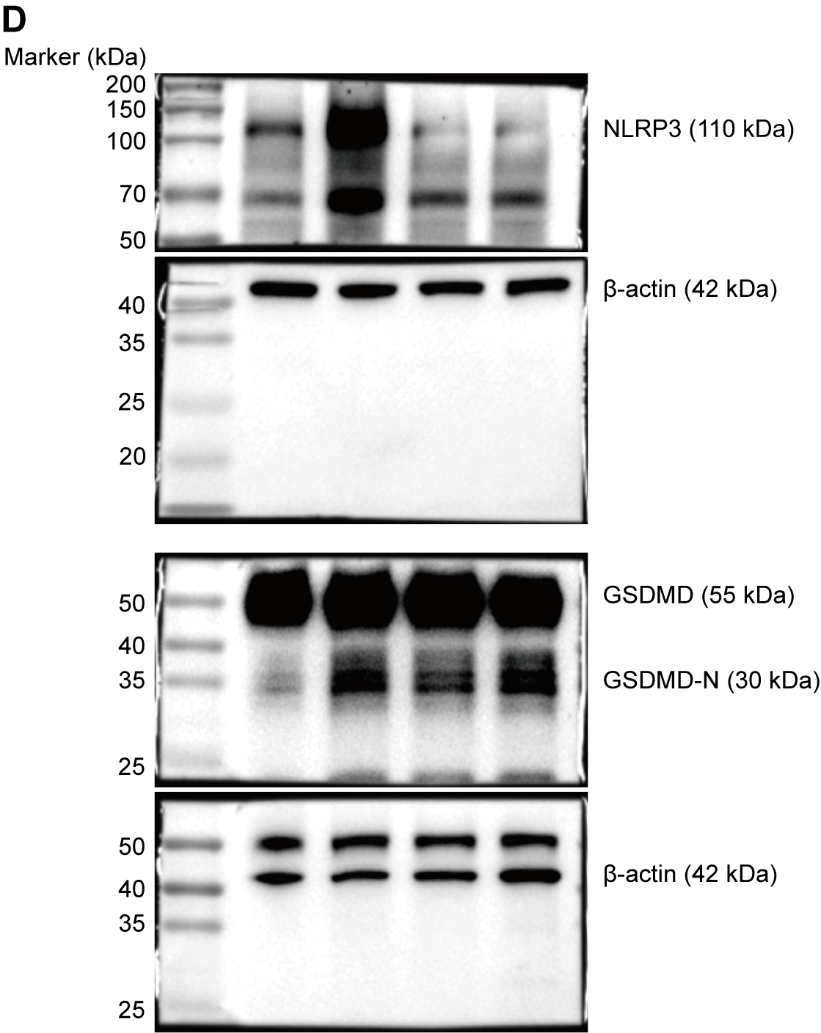


**Fig. S14** The original Western blot images of Fig.7E, G.


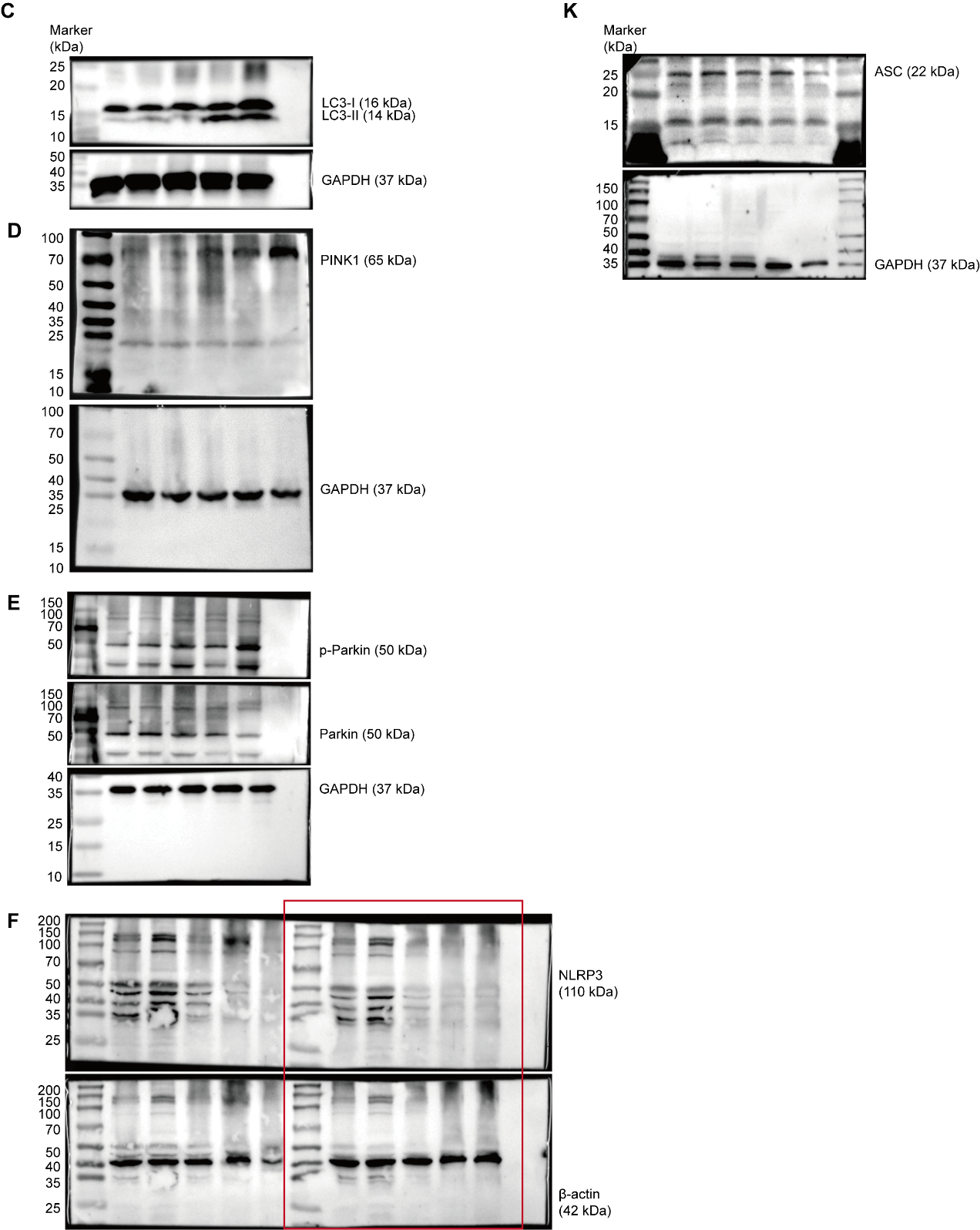


**Fig. S15** The original Western blot images of Fig. 8C, D, E, F, K.


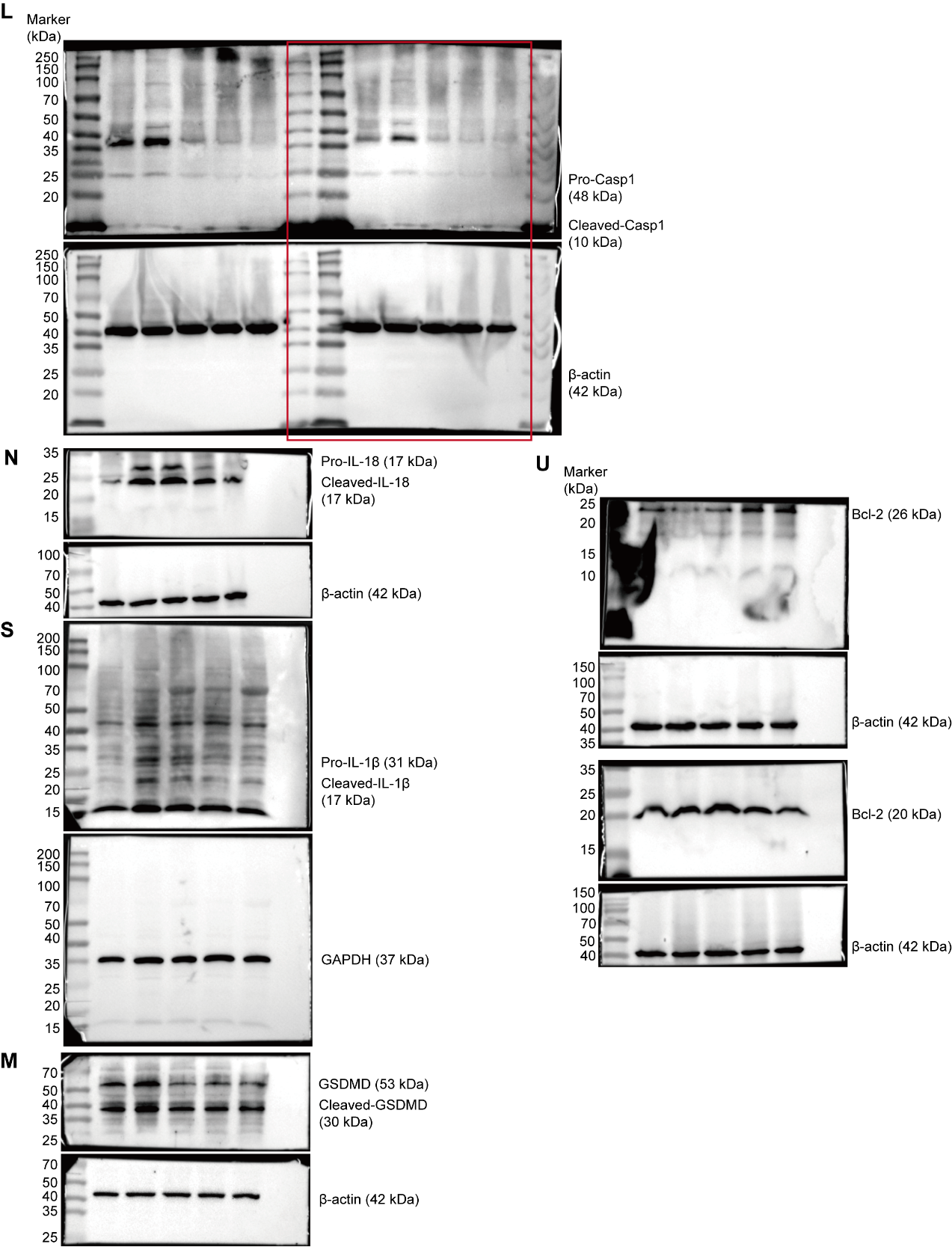


**Fig. S16** The original Western blot images of Fig. 8L, N, S, M, U.
